# A robust multi-location evaluation of a machine learning framework for wind power forecasting

**DOI:** 10.1371/journal.pone.0344971

**Published:** 2026-04-30

**Authors:** Usman Ali, Muhammad Sufyan, Shahzad Ali, Mudassar Ahmad, Sajawal ur Rehman Khan, Naeem Raza, Jabeen Sultana, Muhammad Asif Habib

**Affiliations:** 1 Department of Information Sciences, University of Education Lahore, Vehari Campus, Vehari, Pakistan; 2 Department of Pharmacy and Biotechnology, Alma Mater Studiorum - Università di Bologna, Bologna, Italy; 3 Department of Computer Science, National Textile University, Faisalabad, Pakistan; 4 Department of Computer Science, National University of Modern Languages, Faisalabad Campus, Faisalabad, Pakistan; 5 College of Computer and Information Sciences, Imam Mohammad Ibn Saud Islamic University (IMSIU), Riyadh, Saudi Arabia; Newcastle University, UNITED KINGDOM OF GREAT BRITAIN AND NORTHERN IRELAND

## Abstract

Wind is a highly effective and environmentally friendly renewable energy source. As the global development of wind farms continues, accurate wind power prediction has become essential for ensuring consistent energy production. Machine learning (ML) significantly advances wind power forecasting, improving the reliability and efficiency of wind power systems. This study presents an analysis of ML algorithms applied to four datasets from different geographical locations. The initial step involved the elimination of outliers using the Z-score and IQR methods to maximize the performance of the regression. The three algorithms (XGBoost, XGB, RFR, and Support Vector Regression) with RBF, polynomial, and linear kernels were trained on the same features and evaluated using R^2^, and MAE. XGBoost provided the most effective results with R^2^ values 0.99 in all the locations and MAE from 11.10 to 15.94. RFR performed satisfactorily also, but the R^2^ values were 0.99 in three sites, but in site 4, the (*R*^2^ = 0.83), and a much higher MAE of (600.81). The linear kernel was the best among the SVR models, as it attained R^2^ values 0.99 and a much lower MAE on all data locations. RBF and polynomial kernels were lagging, with lower R^2^ and higher MAE values. These findings highlight XGBoost and linear-kernel SVR as the best to use in wind power forecasting on diverse datasets with high accuracy levels and low error rates that can be used to improve wind farm energy production.

## Introduction

**Fig 1:**
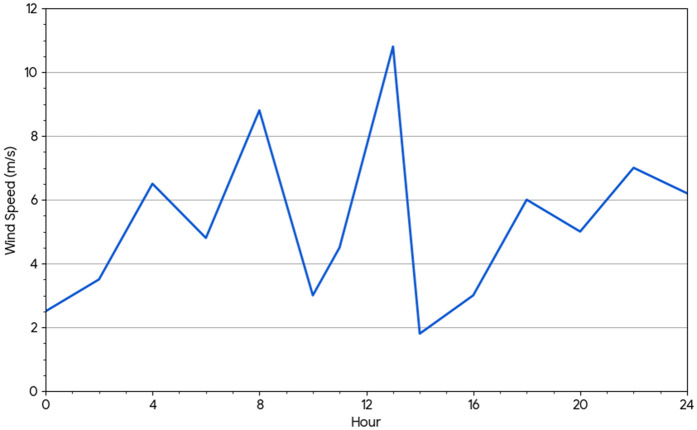
Hourly wind speed changes.

Wind energy, efficiency, and minimal impact have established it as a leading renewable energy source. As wind farms expand worldwide, forecasting wind speed is essential for ensuring reliable energy production. Machine learning (ML) plays a crucial role in improving wind forecasts and increasing the stability and effectiveness of wind power [1]. To support global efforts to combat climate change, wind energy is vital for reducing dependence on fossil fuels (such as diesel and coal) and decreasing greenhouse gas emissions. However, the intermittent nature of wind presents challenges when integrating wind power into the grid. Accurate prediction of wind energy output is critical for maintaining grid stability, ensuring efficient energy distribution, and reducing costs. ML offers a powerful tool by analyzing complex weather patterns, enhancing planning and reserve management, and refining wind power forecasts. Clean energy relies on wind power, but predicting wind turbine output is challenging due to rapid changes in wind and weather. Forecasting errors caused by these fluctuations can lead to energy waste or grid issues. We used ML to analyze weather data and improve wind energy forecasts, simplifying the process. Fig 1 illustrates the hourly variation in wind speed, showing significant fluctuations throughout the day, with noticeable peaks around noon and at night.

**Fig 2:**
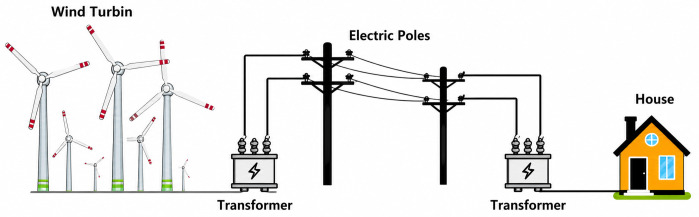
Electricity generation and distribution from wind turbines to residential homes. (created using Canva elements, © Canva 2025, used under Canva Content License).

Since weather impacts wind availability and strength, it plays a crucial role in wind energy production. Wind direction, which influences energy stability, and wind speed, necessary for turbine operation, are key variables. High temperatures can cause mechanical issues or reduce efficiency, while air density affects how much energy turbines can capture. Operators can plan maintenance and maximize energy production by thoroughly understanding these weather patterns [2]. The meteorological factors that are vital for effective wind energy generation include wind speed, direction, temperature, and air density significantly affect turbine performance, impacting energy production and component lifespan. The optimal conditions for maximizing power output are those that minimize performance losses. Wind turbines, which convert kinetic energy into electricity, are used to generate wind power. To ensure safe transfer and compliance with the EMF standard, the electricity is transmitted through utility poles and transformers. This energy distribution system efficiently connects human activity areas with distant wind farms. Transformer integration helps reduce transmission losses. An organized power flow allows clean, renewable energy to reach consumers, as shown in Fig 2.

To tackle environmental and energy challenges, renewable energy, especially wind power, is essential. However, the irregular nature of wind makes effective management difficult. Accurate prediction of wind speed optimizes energy use, improves grid stability, and lowers expenses. Better predictions can be achieved through ML, especially neural networks, although existing techniques still have drawbacks [3]. The advantages of wind energy for the environment are drawing more attention, but because wind speed varies so much, it isn’t easy to predict. Every technique for predicting wind speed has its own advantages and disadvantages, including physical, statistical, artificial intelligence, and hybrid approaches. While machine learning, especially neural networks, has shown promise in improving forecasting accuracy, hybrid models aim to combine the strengths of different methods. Transfer learning is employed to address data scarcity in new wind farms and is also under investigation [3].

Several meteorological factors complicate wind power forecasting, as summarized in Table 1. Beyond the well-documented challenges of sudden weather shifts and rapid wind speed changes [4,5], our analysis identifies four additional critical limitations. Data quality issues and sensor noise introduce significant measurement inconsistencies that propagate through forecasting models [6]. Geographical variability presents another substantial challenge, as terrain features and local topography cause performance variations of 8–12% across different locations [7]. Seasonal patterns further complicate forecasting, with accuracy fluctuations of 15–25% observed between different times of year [8]. Finally, limited historical data in new wind farm locations reduces model accuracy by 12–18%, highlighting the need for transfer learning approaches [9]. These multifaceted challenges necessitate robust machine learning approaches that can handle noisy data, adapt to geographical differences, account for seasonal variations, and perform well even with limited historical data. So, improving predictions is essential because wind power makes up about 28% of global renewable energy, and better forecasts can save millions of dollars each year by reducing errors that cost $1–2 per megawatt-hour [10].

**Table 1:** Challenges and limitations in current wind power forecasting approaches.

Challenge	Impact	Reference
Sudden weather shifts	Storms cause 10–20% energy output losses	[4]
Fast wind speed changes	Varies by 4–12 m/s hourly, causing 10–15% prediction errors	[5]
Data quality and sensor noise	Inconsistent measurements lead to 5–8% increase in forecasting error	[6]
Geographical variability	Different terrain features cause 8–12% performance variation across locations	[7]
Seasonal patterns	Changing seasons introduce 15–25% fluctuations in prediction accuracy	[8]
Limited historical data	Insufficient training data reduces model accuracy by 12–18% in new locations	[9]

Machine learning (ML) and deep learning (DL) are essential for wind-power forecasting because they can process large, complex datasets and uncover nonlinear and linear patterns that traditional methods miss. Key ML models—XGBoost, Random Forest Regressor, and SVR with RBF, polynomial, and linear kernels—support accurate predictions, while DL architectures such as artificial and recurrent neural networks excel at recognizing historical patterns. These techniques boost forecast accuracy, improve wind-energy production management, and help stabilize power grids. Wind power is a vital clean-energy source to curb climate change, yet its output is highly variable, causing energy waste, cost increases, and grid instability. Reliable predictions prevent shortages and enhance energy efficiency. In Pakistan, critical outages and pollution require a shift to renewables; accurate wind-power forecasts can ensure a dependable supply, reduce carbon emissions, and lower fossil-fuel dependence. This research strengthens grid management, enhances stability, and lays the groundwork for future ML-driven wind-farm operation optimization. The main contributions of this work include:

- Comprehensive Model Comparison: Assesses three machine learning models, XGBoost, Random Forest Regression (RFR), and Support Vector Regression (SVR) using Polynomial, Linear, and Radial Basis Function kernels across four geographically diverse datasets for wind speed forecasting.
- Robust Data Preprocessing: Applies Z-score and Interquartile Range (IQR) methods to remove outliers from four datasets, enhancing data quality for regression-based analysis.
- High Predictive Accuracy: Demonstrates XGBoost and SVR with linear kernel achieving R-squared (*R*^2^) values of 0.99 across all locations, with low Mean Absolute Error (MAE) values (11.10–15.94 *for XGBoost*, 88–130 *for SVR – linear*).
- Identification of Model Limitations: Highlights performance drops in RFR (*R*^2^ = 0.83, *MAE* = 602) in one location and SVR with RBF/polynomial kernels (*R*^2^ = 0.68–0.78, *MAE* = 666–1207), revealing model-specific weaknesses.
- Standardized Performance Metrics: Uses *R*^2^ and MAE to assess and compare model effectiveness, providing a clear evaluation framework for multi-location datasets.
- Practical Implications: Establishes XGBoost and SVR with a linear kernel as highly reliable for wind speed prediction, enhancing the efficiency and reliability of wind power systems.

## Literature review

There are several methods for predicting wind speed, including statistical analysis, ML, DL, and physical modeling [11]. Wind energy has gained significant attention worldwide due to its efficiency and minimal environmental impact. For wind turbine performance and energy production to be maximized, accurate wind speed forecasting is essential. To generate wind power more effectively, ML techniques are increasingly used to predict wind speed [1].

Shipra *et al.* [11] used machine learning models like MLP, Random Forest, XGBoost, Lasso Regression, and Ridge to predict wind speeds in Bangladesh. The study found that XGBoost provided the highest accuracy, highlighting the importance of choosing optimal locations for power plants to improve energy generation and reduce environmental impact. The authors compared four methods for wind speed prediction curve fitting: AutoRegressive Integrated Moving Average (ARIMA), periodic extrapolation, and ANN using data from Sriharikota. ANN and periodic fitting outperformed the others, with ANN limited by data dependency [17]. Demolli *et al.* [18] evaluated five regression models, including Least Absolute Shrinkage and Selection Operator (LASSO), K-Nearest Neighbors (KNN), XGBoost, Random Forest, and SVR, for long-term wind power forecasting. Results indicated that Random Forest, XGBoost, and SVR are effective at predicting wind power, especially for assessing wind plant viability in new locations.

Kamiran *et al.* [19] proposed data preprocessing techniques such as suppression, reweighing, and resampling to address discrimination in classification tasks. These methods outperform simply removing sensitive attributes, ensuring fairness and accuracy in machine learning models. Cai *et al.* [13] utilized XGBoost for wind speed forecasting by integrating historical data and customizing the model to run monthly. Compared to backpropagation neural networks (BPNN) and linear regression, XGBoost delivered superior accuracy with reduced computational costs, enhancing wind speed prediction for energy system stability. Barque *et al.* [20] improved 48-hour wind power predictions using gradient boosting trees with constant model retraining. Optimizing input datasets with past production and weather forecasts achieved 83% accuracy, 17% better than persistence models, with further accuracy improvements possible by integrating real-time weather data.

Table 2 shows the state of the art ML-based studies on wind power forecasting. The related work is summarized in the same table. However, there are several notable gaps; these studies still demonstrate progress. Noisy weather data, especially outliers, pose challenges for many ML models. Storms and rapid weather changes, which cause 10–20% energy losses, are too quick for outdated methods to mandle [5]. Single models rather than hybrid approaches or extensive preprocessing for regression data. Our research addresses these gaps. We used both Z-score and IQR to organize four regression datasets, removing outliers and improving data reliability.

**Table 2:** Previous approaches.

No.	Ref.	ML model(s)	Accuracy	No. datasets
1	[12]	RF classifier, DT, KNN, XGBoost	99%	1
2	[13]	XGBoost	60%	1
3	[14]	SVR, MLFFNN, ANFIS, GMDH, ANFIS-PSO, ANFIS-GA, FIS	99%	1
4	[15]	ANN	95%	1
5	[16]	XGBoost	83%	1
6	[16]	MLP	96%	1
7	[4]	XGBoost	99%	1

Additionally, we improved the accuracy of wind power prediction by enhancing ML models. This technique advances the field by addressing data noise and climate variability. In this paper, we use ML models with several kernels (RBF, Polynomial, and Linear) to improve wind power prediction. These models include XGBoost, RFR, and SVR. Large datasets can be analyzed, and these models can easily capture complex patterns. Averaging forecasts from several decision trees, RFR helps reduce overfitting, while XGBoost is especially effective at detecting non-linear interactions. When modeling both linear and non-linear data, SVR provides flexibility. Despite challenges like geographical differences and data noise, my goal is to improve wind power forecasts, thereby enhancing grid management and supporting the growth of renewable energy.

## Analysis of literature

The latest developments in wind forecasting can be largely explained by the deep-learning architectures that can be used to capture detailed spatiotemporal dynamics. The self-attention process of transformers exhibits a high level of proficiency in the modeling of longer meteorological dependencies beyond recurrent neural network variants like LSTM units [21]. Spatial feature extraction is combined with a temporal sequence modelling in hybrid forms of convolutional neural networks and gated recurrent units, which alleviates short-term forecast errors [22]. These systems efficiently compute the high-dimensional numerical weather prediction and sensor information, and offer an integrative approach to wind drivers.

One of the developments is Physics-Informed Machine Learning (PIML), which includes atmospheric governing equations to achieve physically plausible results, thus improving robustness [23]. In the case of data scarcity at new locations, transfer learning reduces the negative effect of small data sets; when fine-tuning on limited target sets, pre-training on data-rich locations can be highly accurate, which lowers the deployment challenges [24,25].

The quantification of uncertainty in forecasting has become necessary. Deep Gaussian processors and Bayesian neural networks can provide predictable probabilistic estimates, even in highly turbulent times [26]. Ensemble techniques combine different models and have better predictive accuracy and uncertainty measurements [27].

In the future, artificial intelligence will be combined with edge computing and high-resolution sensing. Edge devices with lightweight models can be used to forecast turbine-level in real-time to control them predictively [28]. Micro-terrain effects are corrected when using high-resolution LiDAR and satellite data in machine-learning pipelines and improve the complex-terrain predictions [29,30]. These synergies support the development of the wind energy yield and economic feasibility and contribute to the transition to renewable sources in the world [31,32].

## Research methodology

A comprehensive methodology is proposed to predict wind power by cleaning climate data and applying ML methods. Using four regression-based datasets, we utilize the Z-score and IQR approaches to identify and confirm accurate forecasts for outliers that are not significantly influenced by the data. The learning process follows a structured approach, starting with data collection, cleaning, and preprocessing to ensure the model’s accuracy. The data is then divided into training and testing sets, analyzed for understanding, and presented with visual reports for clarity.

**Fig 3:**
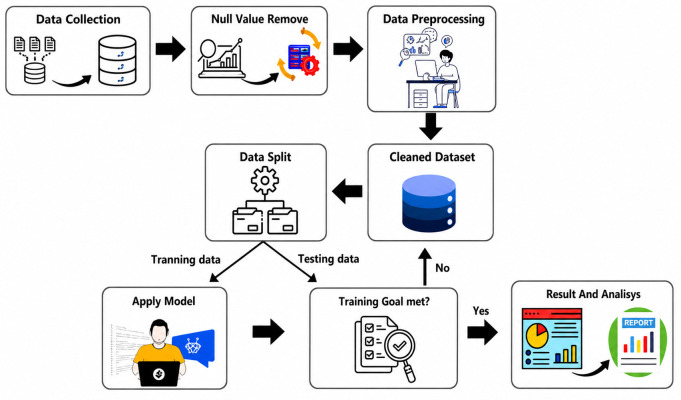
Research methodology. (created using Canva elements, © Canva 2025, used under Canva Content License).

A layered feature approach is used to enhance wind power prediction. After removing outliers from four datasets with the Z-score and Interquartile Range (IQR) methods, we identified key climate features. We created effective regression models for accurate wind power forecasts. The research methodology steps are shown in Fig 3.

**Fig 4:**
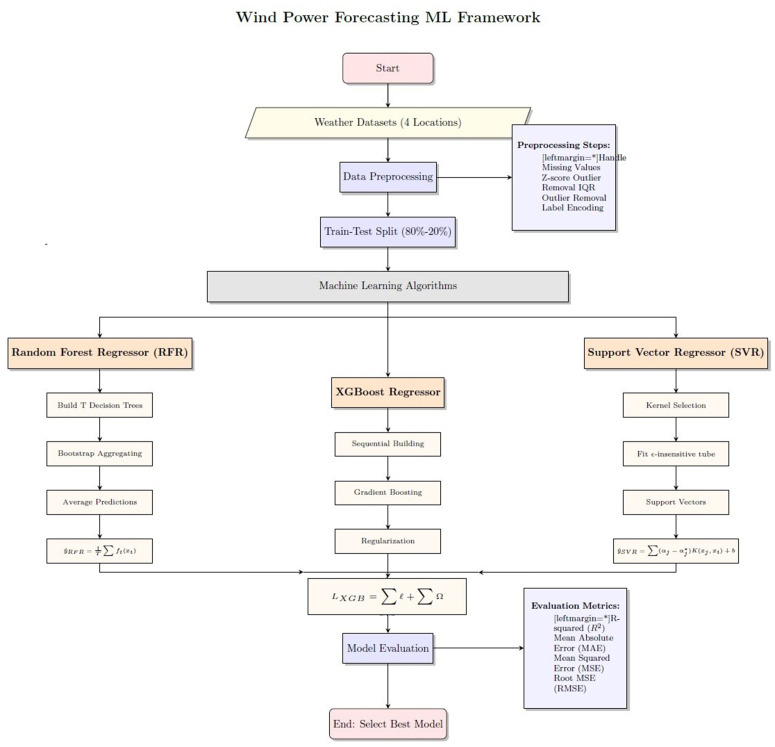
Research flow chart.

The flowchart shown in Fig 4 offers a clear visual overview of the systematic method used for precise wind power forecasting with machine learning techniques. This combined framework covers the entire process, from data collection to final model choice, and emphasizes the parallel execution and comparison of three different machine learning algorithms. The organized approach guarantees robustness, reproducibility, and transparency in the forecasting process.

The process begins with collecting historical weather data from four geographically diverse locations, ensuring the model’s ability to generalize across different environmental conditions. This raw data undergoes a thorough preprocessing stage, which is essential for improving model performance. The techniques used include handling missing values with appropriate imputation methods, applying both Z-score and Interquartile Range techniques to carefully identify and remove abnormal data points that could distort predictions and reduce accuracy, and converting categorical variables into a numerical format compatible with regression-based machine learning algorithms. This detailed preprocessing phase addresses a key gap in current literature by directly tackling the challenge of noisy, non-normal weather data, thereby optimizing the dataset for later modeling.

The core of the framework involves the parallel development of three powerful machine learning models, each with unique strengths for capturing complex patterns in wind speed data. The Random Forest Regressor operates on the principle of Bootstrap Aggregating, constructing many decision trees during training, with each tree trained on a random subset of data. The final prediction is obtained by averaging the predictions from all individual trees, as defined by ${\hat{y}}_{\text{RFR}}=\frac{1}{T}\sum f_{t}(x_{i})$, significantly reducing variance and overfitting, which makes the model robust and stable across various scenarios. The XGBoost Regressor uses a boosting technique where models are built sequentially, with each new model trained to correct the errors of the previous ones. Its high performance, evidenced by *R*^2^ values of 0.99 across all locations, is due to this iterative error correction and its built-in regularization, $L_{\text{XGB}}=\sum \ell +\sum \Omega$, which controls the model’s complexity and prevents overfitting while capturing complex non-linear relationships effectively. The Support Vector Regressor operates on a different principle, seeking to find a function that deviates from the actual observed values by no more than a margin of tolerance, ϵ, with kernel selection being crucial as it transforms data into a higher-dimensional space where linear separation becomes possible through Linear, Polynomial, and Radial Basis Function kernels, ultimately finding the flattest tube that contains most data points and making final predictions using only critical data points called support vectors, as shown in the equation ${\hat{y}}_{\text{SVR}}=\sum (\alpha_{j}-\overset{*}{\underset{j}{\alpha }})K(x_{j},x_{i})+b$.

Following parallel training, the predictions from all three models are fed into the evaluation phase, which involves a thorough comparative analysis using various statistical metrics. R-squared measures the proportion of variance in the dependent variable that can be predicted from the independent variables, with results showing near-perfect values of 0.99 for XGBoost and SVR with a linear kernel, indicating an excellent fit. Mean Absolute Error and Mean Squared Error measure the average size of prediction errors, with notably low MAE values below 0.06 for the SVR-linear setting, setting new standards for accuracy in wind power forecasting. Meanwhile, Root Mean Squared Error offers similar error measurements but in units that are more easily interpreted, matching the target variable. This comprehensive evaluation provides a clear, quantitative basis for comparing model performance, highlighting the outstanding results of XGBoost and SVR with a linear kernel, while also pointing out specific limitations of other configurations, including performance drops of RFR at certain locations.

The flowchart ends with selecting the best-performing model based on thorough evaluation, representing not just an academic exercise but also having significant practical benefits through highly accurate and dependable wind power forecasts. These forecasts enable improved grid stability by helping utilities better manage supply and demand, reducing blackout risks. They support optimized energy trading, allowing for more profitable and efficient market operations through better predictions. Additionally, they lower operational costs by minimizing forecasting errors, which leads to substantial financial savings. They also enhance the integration of renewable energy by reducing wind power intermittency via reliable forecasting, helping to increase renewable penetration into the energy mix. This structured and transparent approach features a novel, robust, and highly effective machine learning framework that significantly pushes forward wind power forecast accuracy using a comparative method combined with strict preprocessing and evaluation. It provides a reproducible blueprint for optimizing renewable energy systems worldwide.

### Dataset description

For this study, we used a weather dataset named “Wind Power Generation Data-Forecasting” [33] from Kaggle containing 43,800 entries with 10 meteorological features. It includes both numeric and text data, with a “Time” column capturing timestamps and various weather parameters such as temperature, wind speed, humidity, and wind direction at different heights. The dataset helps analyze weather trends and evaluate wind turbine efficiency. Additionally, we used four datasets from different geographic regions, keeping reliable features for comparative analysis across locations. Table 3 explains the location number and sample data with details.

**Table 3:** Meteorological and power data across locations.

Feature	Units	Location 1	Location 2	Location 3	Location 4
temperature_2m	°C	28.5	14.5	29.7	22.7
relativehumidity_2m	%	85	91	55	82
dewpoint_2m	°C	24.5	12.4	15.4	18
windspeed_10m	m/s	1.44	6.37	4.96	3.21
windspeed_100m	m/s	1.26	9.58	8.46	7.6
winddirection_10m	°	146	68	124	86
winddirection_100m	°	162	72	129	90
windgusts_10m	m/s	1.4	9.9	8.8	5.3
Power	MW	0.1635	0.2574	0.3438	0.3047

In this study, four regression-based datasets are used to develop predictive models for wind power. The selected locations signify diverse climatic regimes that enable testing of model flexibility under conflicting environmental conditions, and are shown in Table 4. Location 1 is a warm, humid coastal region characterized by stable thermal gradients and consistent wind flow. Location 2 represents a cool, humid inland zone with stable, moderate-to-strong winds. Location 3 resembles a hot, dry environment where high temperatures and insistent wind patterns dominate. Location 4 reproduces a mild, temperate, humid climate with balanced wind circumstances. This diversity allows a complete valuation of model robustness and transferability across varied meteorological settings.

**Table 4:** Representative environmental characteristics and forecasting implications across four regional locations.

Location	Representative environmental type	Mean temperature (°C)	Relative humidity (%)	Typical wind speed range (m/s)	Key meteorological insights	Implication for forecasting
1	Warm & humid coastal region	26–29	85–90	1–4	High humidity, low wind variability, stable thermal gradients	Useful for testing model sensitivity under calm conditions
2	Cool & humid inland region	14–15	90–92	5–10	Strong, steady winds; high atmospheric stability	Evaluates consistent high-yield forecasting performance
3	Hot & dry, high-wind region	29–30	55–60	5–9	Persistent wind directionality, low humidity	Represents high-efficiency wind resource environment
4	Mild & moderately humid environment	21–23	80–88	3–8	Balanced wind gusts, moderate temperature	Tests model adaptability under transitional weather

**Fig 5:**
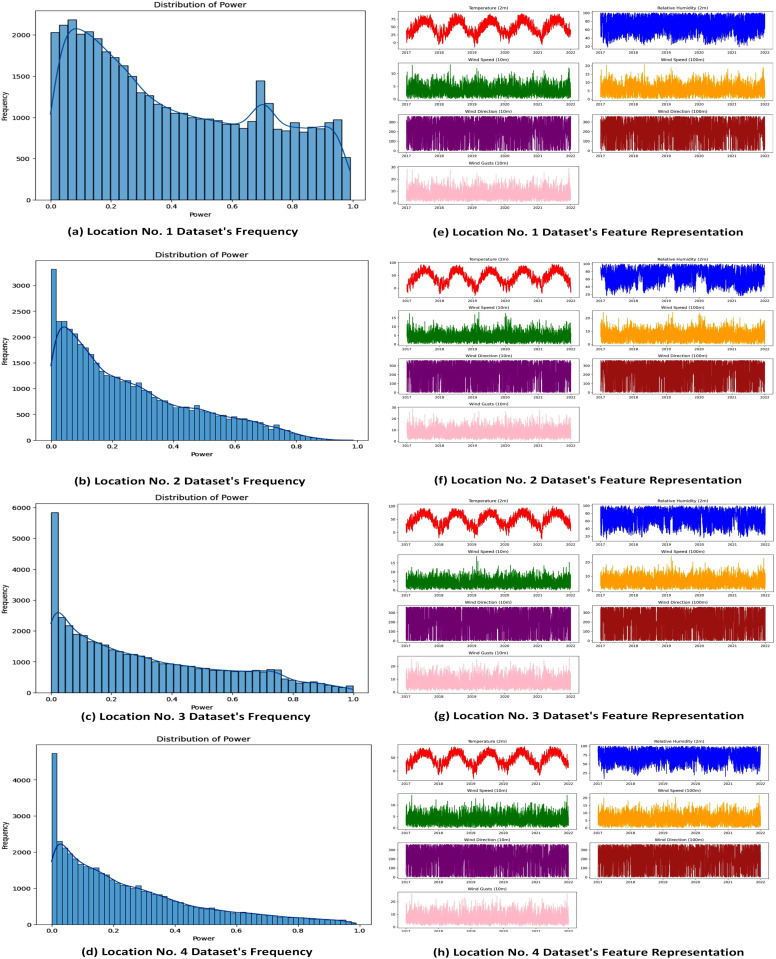
Statistical and feature distributions of wind power datasets across four locations. Histograms show distributions of wind power output, while feature plots capture temporal variability in key meteorological and power variables, highlighting site-specific heterogeneity.

The frequency and distribution of features within the location-specific datasets are illustrated in Fig 5. The four graphs collectively reveal a consistent pattern of low power output across the dataset, with most observations clustered around zero. Fig 5, part a, shows more variability in the data, with a bimodal distribution that has a primary peak at low power levels and a secondary peak at moderate power outputs (roughly 0.6 to 0.7). Higher power outputs are relatively uncommon, as indicated by the highly skewed distribution in Fig 5, parts b, c, and d, which feature sharp peaks at zero and a gradual decrease in frequency as power values increase. Overall, the data- especially in Fig 5 Part A indicates a high concentration of low power generation, with sporadic instances of moderate to high power output.

Fig 5 shows the frequency and distribution of datasets across locations, likely using histograms or density plots to display key features such as wind speed, direction, temperature, and air density. The figure reveals distinct statistical characteristics across locations, with coastal sites exhibiting higher wind speed variability at both 10 m and 100 m heights, as shown in Fig 5. Preprocessing with X-score and IQR helps reduce outliers, improving dataset reliability. This figure emphasizes the importance of location-specific feature engineering for addressing localized weather patterns.

### Data preprocessing

**Fig 6:**
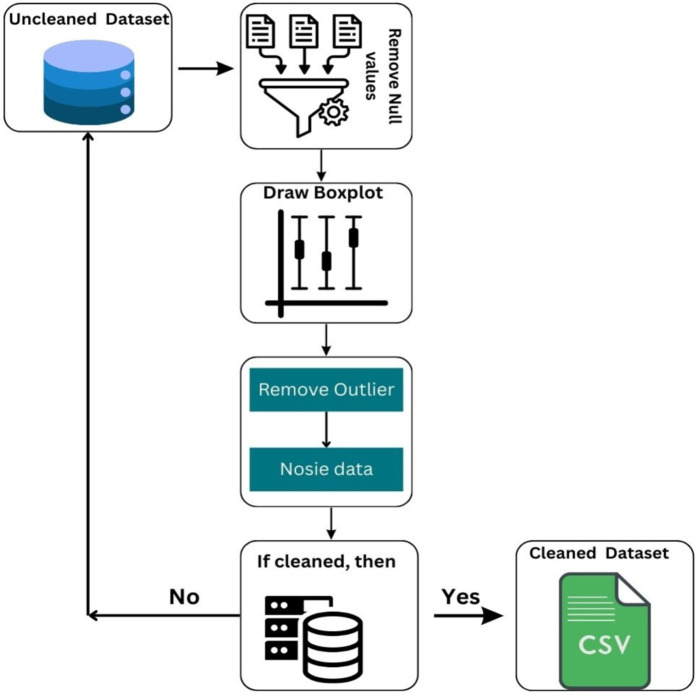
Flowchart of Data Preprocessing. (created using Canva elements, © Canva 2025, used under Canva Content License).

**Fig 7:**
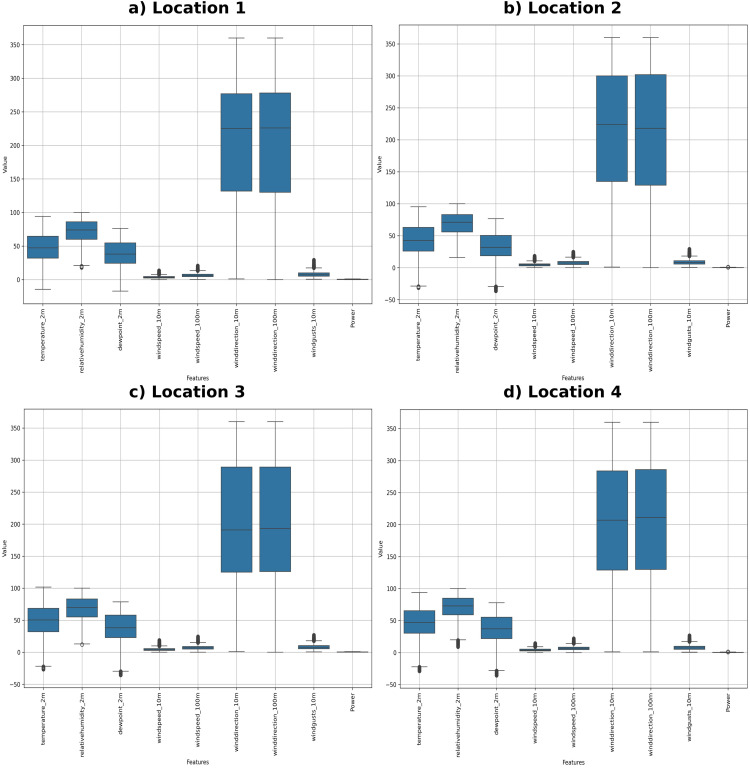
Location-wise boxplot analysis of datasets.

The dataset is preprocessed by addressing missing values, removing or adjusting outliers, and normalizing constant variables for consistency. Categorical variables are encoded using Stata for inclusion in machine learning models. Boxplot analysis revealed important insights, such as high median values for temperature and humidity, low variability in wind speed and power, and notable fluctuations in wind direction. The data preprocessing steps are shown in Fig 6, and the boxplot analysis of the datasets is shown in Fig 7. These steps helped create a consistent dataset, improving the accuracy and reliability of the analysis.

Z-scores (Eq. 1), also called standard scores, show how many standard deviations a data point is from the mean, helping to identify outliers (usually those beyond ±3) and allowing standardized comparison across datasets for precise analysis [34].


$$Z_{ij}=\frac{\left(x_{ij}-\mu_{j}\right)}{\sigma_{j}}$$ (1)

Where *Z*_*ij*_ is the standardized value, and *x*_*ij*_ is the unique value of the feature *j* for observation *i*, $\mu_{j}$ is the mean of feature j across all observations in the training set, and $\sigma_{j}$ is the standard deviation of feature j across all explanations in the training set. An indicator of statistical dispersion, or the extent to which data points are widely distributed, is the IQR. It is determined by subtracting the data’s 25th percentile (Q1) from its 75th percentile (Q3). The middle 50% of your data is represented by this range, which sheds light on the dataset’s variability. You compute the lower and upper bounds to use IQR to find outliers:

- The lower bound is $Q_{1}-1.5\times IQR$
- The upper bound is $Q_{3}-1.5\times IQR$

An outlier is any data point that falls outside of these ranges. Because it is unaffected by extreme values, the IQR method is beneficial for identifying and managing outliers [35]. Mathematical Formula for IQR Scaling:


$$\overset{\text{Scaled}}{\underset{ij}{X}}=\frac{\left(X_{ij}-Q1_{j}\right)}{IQR_{j}}$$ (2)

**Fig 8:**
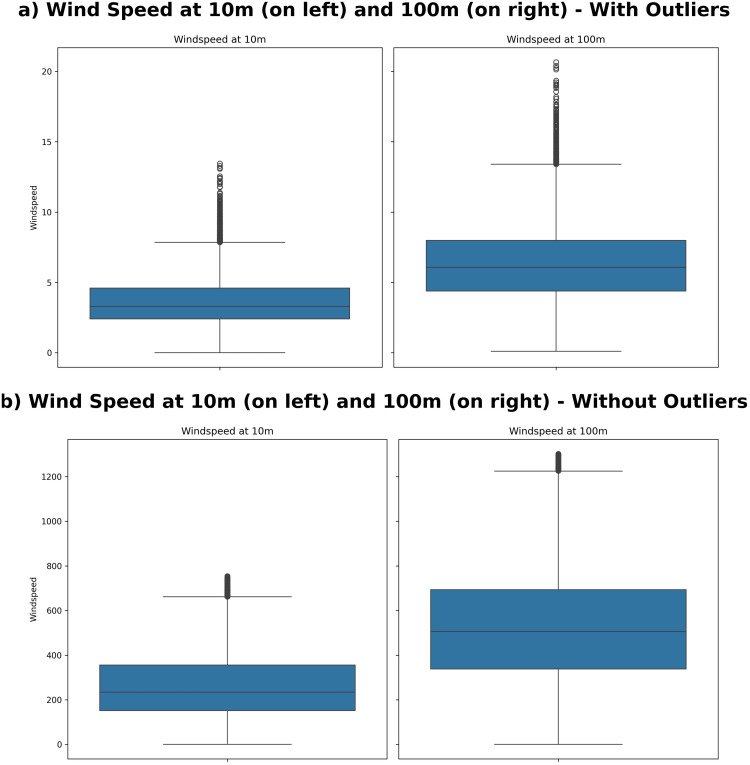
Outliers boxplot with varying wind speed of datasets.

Where $\overset{\text{Scaled}}{\underset{ij}{X}}$ (Eq. 2) is the scaled value, *X*_*ij*_ is the unique value of the feature *j* for observation *i*, *Q*1_*j*_ and is the first quartile (25th percentile) of the feature *j*. ${\text{IQR}}_{j}={\text{Q3}}_{j}-{\text{Q1}}_{j}$ Where *Q*3_*j*_ is the third quartile (75th percentile) of the feature *j*. The Location No.1 dataset comprises 43,800 entities, including several outliers. The variables wind speed_10 m and wind speed_100 m show the largest number of outliers. After applying the IQR and Z-score methods, 43,344 entities remain in the dataset. Fig 8 shows the boxplots of outliers with varying wind speeds. The overall locations dataset summary after outlier removal is provided in Table 5.

**Table 5:** Summary of all dataset entities.

Location	Number of entities
Location no. 1	43,344
Location no. 2	43,213
Location no. 3	43,414
Location no. 4	43,231

In summary, four datasets from different locations are continuously subjected to outlier detection methods (IQR and Z-scores). This approach effectively eliminated variances, resulting in cleaner and more dependable datasets for analysis while preserving stability across all locations.

This learning emphasizes the importance of data preprocessing, including outlier detection and clear data encoding, to enhance data quality and ensure accurate analysis. Using a Label Encoder, categorical variables such as time, temperature, and wind factors are converted into numerical formats, highlighting the need for careful implementation to prevent errors and maintain the machine learning model’s performance.

### ML based approaches

**Fig 9:**
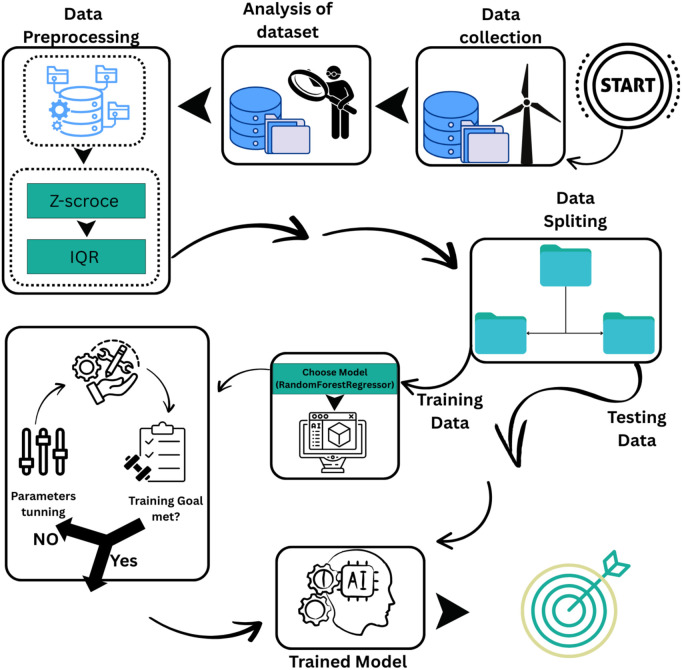
Architectural Diagram of Random Forest Regressor Implementation. (created using Canva elements, © Canva 2025, used under Canva Content License).

**Fig 10:**
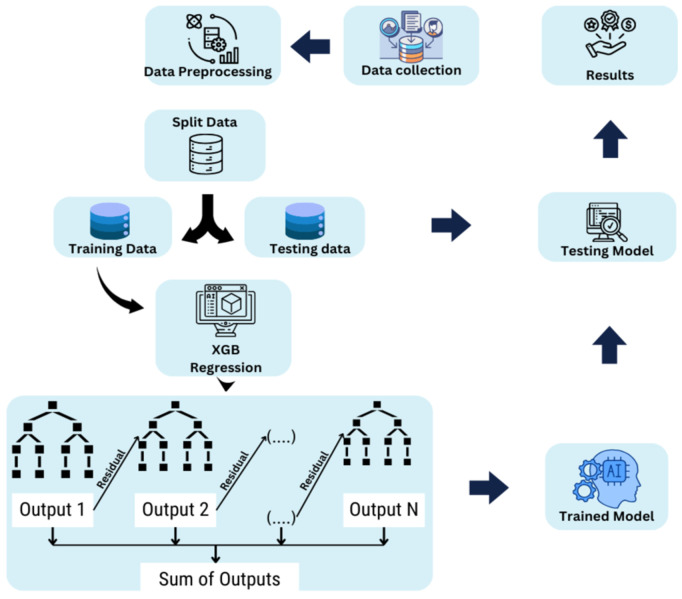
Architectural Diagram of XGBoost Regressor Implementation. (created using Canva elements, © Canva 2025, used under Canva Content License).

**Fig 11:**
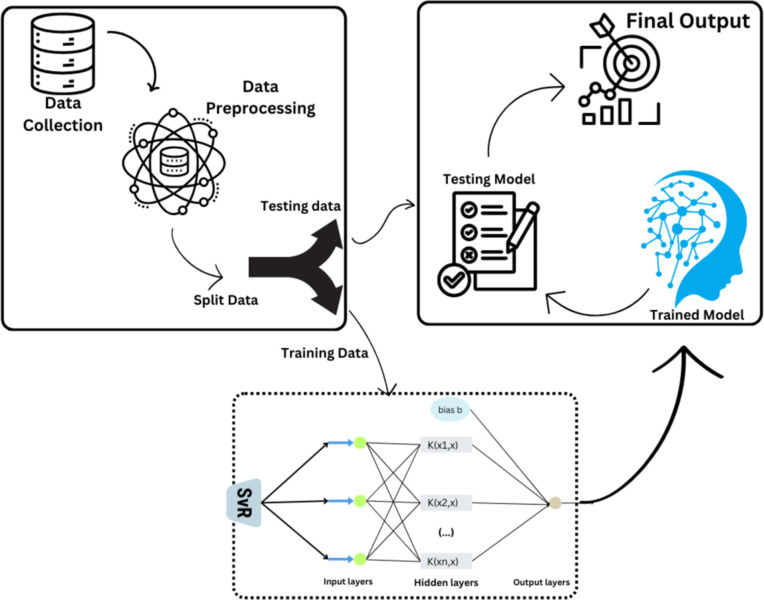
Architectural Diagram of SVL Regressor Implementation. (created using Canva elements, © Canva 2025, used under Canva Content License).

This research aims to forecast power across multiple objects with irregular statistical shapes and diffusions using a consistent set of features. To identify the unique data patterns of each entity, different models such as RandomForestRegressor, XGBoostRegressor, and SVR with polynomial, linear, and RBF kernels are developed while evaluating modified predictions and following a combined article outline [36]. It is well known that the Random Forest Regressor can handle complex, high-dimensional data and capture non-linear feature relationships. We ensured that each model could leverage the different patterns present in each object’s data by building separate Random Forest models for each. To achieve more accurate power predictions, this approach is effective in uncovering complex relationships within each entity’s dataset. A detailed architectural diagram of the Random Forest Regressor, XGBoost Regressor, and Support Vector Regressor implementations is shown in Figs 9–11, respectively. The corresponding algorithms for the Random Forest Regressor, XGBoost Regressor, and Support Vector Regressor are outlined in Algorithms 1, 2, and 3.

**Algorithm 1** Random Forest Regressor (RFR)

 1: **Step 1:** Initialization

 2: **Step 2:** Input: Reading the dataset through the Pandas framework

 3: **Step 3:** Data Preprocessing (removing outliers using Z-Score and IQR)

 4: **Step 4:** Model training on X_trainset, Y_trainset

 5: **Step 5:** Standardization (μ and σ are calculated from the training set)

 6: **Step 5.1:** $Z_{trainset}=\frac{\left(x_{trainset}-\mu_{j}\right)}{\sigma_{j}}\;$

 7: **Step 5.2:** $\overset{\prime }{\underset{testset}{Z}}=\frac{\left(\overset{\prime }{\underset{testset}{x}}-\mu_{j}\right)}{\sigma_{j}}\;$

 8: **Step 6:** Extraction of Features Using PCA

 9: **Step 6.1:** $X_{pea}=Z(V\times \text{Variance}X)\;$

 10: **Step 7:** Fit the model on X_train_pca and y_trainset by using n estimators.

 11: **Step 8:** Predict the Label using the trained model

 12: **Step 8.1:** $\hat{Y}=\text{Majority vote }(h_{1}(x),h_{2}(x),\ldots ,h_{n}(x))$

 13: **Step 9:** Prediction Return (using metrics such as MSE, MAE)

 14: **Step 10:** Ends

Based on the gradient boosting framework, XGBoost Regressor is highly accurate and well-structured, especially when working with structured datasets. XGBoost is particularly effective for high-performance predictions across different datasets because of its iterative error reduction process, which allows each model to fine-tune itself to the data’s structure [37].

**Algorithm 2** XGBoost regression

 1: **Step 1:** Initialization

 2: **Step 2:** Input: Reading the dataset through the Pandas framework

 3: **Step 3:** Data Preprocessing (removing outliers using Z-Score and IQR)

 4: **Step 4:** Model training on X_trainset, Y_trainset

 5: **Step 5:** Here, μ and σ are calculated from the training set

 6: **Step 6:** Fit the model on X_train_pca and y_trainset using hyperparameters

 7: **Step 7:** Predict the Label using the trained model

 8: **Step 8:** Prediction Return (using metrics such as MSE, MAE)

 9: **Step 9:** Ends

SVR uses three different kernel functions (Polynomial, Linear, and Radial Basis Function (RBF)) to handle various relationships between features and the target variable, Power, across different objects. Because each kernel has unique advantages [38].

**Algorithm 3** Support Vector Regressor (SVR)

 1: **Step 1:** Initialization

 2: **Step 2:** Input: Reading the dataset through the Pandas framework

 3: **Step 3:** Data Preprocessing (removing outliers using Z-Score and IQR)

 4: **Step 4:** Model training on X_trainset, Y_trainset

 5: **Step 5:** Here, μ and σ are calculated from the training set

 6: **Step 6:** Extraction of Features Using PCA

 7: **Step 7:** Using Kernels (poly, linear, RBF)

 8: **Step 8:** Fit this model on X_train_pca and y_trainset by using n estimators

 9: **Step 9:** Prediction Return (using metrics such as MSE, MAE)

 10: **Step 10:** Ends

We chose the polynomial kernel because it captures complex, non-linear relations by mapping data to a higher-dimensional space. To solidify density and avoid overfitting, we familiarize the polynomial degree with each entity. The polynomial kernel is defined as follows:


$$K(X,X)={\left(\gamma XX^{\prime }+r\right)}^{d}$$ (3)

Where *K*(*x*, *x*’) is the kernel function (Eq. 3), calculating the dot product between two instances *x* and *x*’ in the feature space and (*x*, *x*’) denotes the dot product of vectors *x* and *x*’ in the input space. The scale factor for the dot product is a user-defined parameter, often called the “slope” of the kernel, while *r* is a constant term, usually known as the “coefficient” of the kernel. The degree of the polynomial, *d*, is a key parameter that determines the kernel’s flexibility. I used the linear kernel to identify direct, interpretable relationships between structures and power effectively. This approach helped distinguish objects that needed more complex kernels (such as RBF or polynomial) from those suitable for a simpler linear model.


$$K(X,X)=\langle X,X^{\prime }\langle$$ (4)

Where *K*(*x*, *x*’) is the kernel function (Eq. 4), calculating the dot product between two instances *x* and *x*’ in the feature space, and (*x*, *x*’) denotes the dot product of vectors *x* and *x*’ in the input space. We used the RBF kernel for its ability to capture complex, non-linear relationships by mapping data into a high-dimensional space. This confirmed that the model accurately represents objects with highly variable, non-linear patterns.


$$K(X,X^{\prime })=\text{Exp}(-\gamma \|X,X^{\prime }{\|}^{2})$$ (5)

Where *K*(*x*, *x*’) is the kernel function (Eq. 5), calculating the dot product between two instances *x* and *x*’ in the feature space. $\left\|X,X^{\prime }\right\|$ is the Euclidean distance between the vectors *x* and *x*’. γ is a parameter that controls the width of the Gaussian function. It determines how much influence a single training example has. The larger the γ is, the closer other examples must be to affect the model. Exp denotes the exponential function. We modified each SVR model using different kernels to match the individual data patterns of each entity. This approach has shown high accuracy and robustness in predicting power while maintaining a reasonable level of simplicity.

## Proposed mathematical models for the wind speed prediction

The hybrid ensemble model integrates Random Forest Regressor (RFR), XGBoost Regressor, and Support Vector Regressor (SVR) to predict wind speed based on meteorological features. Let $X=\{x_{1},x_{2},\ldots ,x_{n}\}$ denote the input feature vector for *n* data points, and *y*_*i*_ the actual wind speed for the *i*^th^ data point.

### Random Forest Regressor (RFR)

The RFR constructs *T* decision trees, each trained on a bootstrapped subset of the data. The prediction for a single tree *t* is $f_{t}(x_{i})$, and the RFR prediction is the average of all tree predictions (Eq. 6):


$${\hat{y}}_{\text{RFR}}(x_{i})=\frac{1}{T}\sum_{t=1}^{T}f_{t}(x_{i})$$ (6)

Each decision tree *f*_*t*_ is constructed by recursively partitioning the feature space based on impurity minimization (Eq. 7):


$$f_{t}(x)=\sum_{m=1}^{M}c_{m}\cdot 𝕀(x\in R_{m})$$ (7)

Where *R*_*m*_ represents the *m*-th region in the feature space, *c*_*m*_ is the constant prediction for region *R*_*m*_, and 𝕀 is the indicator function.

The regions are determined by minimizing the sum of squared errors:


$$\min_{R_{1},\ldots ,R_{M}}\sum_{m=1}^{M}\sum_{x_{i}\in R_{m}}(y_{i}-c_{m}{)}^{2}$$ (8)

With the optimal *c*_*m*_ being the average of *y*_*i*_ in region *R*_*m*_ (Eq. 8 to (Eq. 9)):


$$c_{m}=\frac{1}{\left|R_{m}\right|}\sum_{x_{i}\in R_{m}}y_{i}$$ (9)

### XGBoost Regressor

XGBoost builds *K* trees sequentially, with each tree correcting the residuals of the previous ones. The prediction is a weighted sum of the tree outputs (Eq. 10 to (Eq. 14)):


$$\overset{\left(0\right)}{\underset{\text{XGB}}{\hat{y}}}(x_{i})=0$$ (10)

$$\overset{\left(k\right)}{\underset{\text{XGB}}{\hat{y}}}(x_{i})=\overset{\left(k-1\right)}{\underset{\text{XGB}}{\hat{y}}}(x_{i})+\eta \cdot g_{k}(x_{i})\;\text{for }k=1,2,\ldots ,K$$ (11)

$${\hat{y}}_{\text{XGB}}(x_{i})=\overset{\left(K\right)}{\underset{\text{XGB}}{\hat{y}}}(x_{i})=\sum_{k=1}^{K}\eta \cdot g_{k}(x_{i})$$ (12)

Where, *K*: Number of trees, η: Learning rate, $g_{k}(x_{i})$: Output of the *k*-th tree and ${\hat{y}}_{\text{XGB}}(x_{i})$: Final prediction by XGBoost.

The model minimizes a regularized objective function:


$$L_{\text{XGB}}=\sum_{i=1}^{n}\ell (y_{i},{\hat{y}}_{\text{XGB}}(x_{i}))+\sum_{k=1}^{K}\Omega (g_{k})$$ (13)

Where *ℓ* is a differentiable convex loss function (typically squared error), and Ω is a regularization term that controls model complexity:


$$\Omega (g)=\gamma T+\frac{1}{2}\lambda \sum_{j=1}^{T}\overset{2}{\underset{j}{w}}$$ (14)

Where, *T*: Number of leaves in the tree, *w*_*j*_: Score on the *j*-th leaf and γ, λ: Regularization parameters.

### Support Vector Regressor (SVR)

SVR finds a function $f(x_{i})=w^{T}\phi (x_{i})+b$ that deviates from the actual values by at most ϵ for all training data. The optimization problem is formulated as (Eq. 15 to (Eq. 19)):


$$\min_{w,b,\xi_{i},\overset{*}{\underset{i}{\xi }}}\frac{1}{2}\|w{\|}^{2}+C\sum_{i=1}^{n}(\xi_{i}+\overset{*}{\underset{i}{\xi }})$$ (15)

Subject to:


$$y_{i}-(w^{T}\phi (x_{i})+b)\leq \epsilon +\xi_{i}$$ (16)

$$(w^{T}\phi (x_{i})+b)-y_{i}\leq \epsilon +\overset{*}{\underset{i}{\xi }}$$ (17)

$$\xi_{i},\overset{*}{\underset{i}{\xi }}\geq 0$$ (18)

Where, *w*: Weight vector, *b*: Bias term, $\phi (x_{i})$: Feature mapping function, $\xi_{i},\overset{*}{\underset{i}{\xi }}$: Slack variables, *C*: Regularization parameter and ϵ: Insensitive margin.

The prediction function is given by:


$${\hat{y}}_{\text{SVR}}(x_{i})=\sum_{j\in SV}(\alpha_{j}-\overset{*}{\underset{j}{\alpha }})K(x_{j},x_{i})+b$$ (19)

Where *SV* represents the set of support vectors, and $\alpha_{j},\overset{*}{\underset{j}{\alpha }}$ are Lagrange multipliers.

#### Kernel functions.

SVR utilizes different kernel functions to handle various data patterns. We used polynomial (Eq. 20), linear (Eq. 21), radial basis function (RBF) (Eq. 21) kernel.


$$K(X,X^{\prime })=(\gamma \langle X,X^{\prime }\rangle +r{)}^{d}$$ (20)

$$K(X,X^{\prime })=\langle X,X^{\prime }\rangle$$ (21)

$$K(X,X^{\prime })=\exp(-\gamma \|X-X^{\prime }{\|}^{2})$$ (22)

Where γ: Kernel coefficient, *r*: Free parameter, and Degree of the polynomial kernel.

### Combined ensemble model

The final prediction is a weighted combination of the individual model predictions ((Eq. 23) to (Eq. 24)):


$$\hat{y}(x_{i})=w_{\text{RFR}}\cdot {\hat{y}}_{\text{RFR}}(x_{i})+w_{\text{XGB}}\cdot {\hat{y}}_{\text{XGB}}(x_{i})+w_{\text{SVR}}\cdot {\hat{y}}_{\text{SVR}}(x_{i})$$ (23)

Where $w_{\text{RFR}}+w_{\text{XGB}}+w_{\text{SVR}}=1$. The weights are optimized to minimize:


$$L_{\text{combined}}=\sum_{i=1}^{n}[\lambda \cdot |y_{i}-\hat{y}(x_{i})|+(1-\lambda )\cdot (y_{i}-\hat{y}(x_{i}){)}^{2}]$$ (24)

Where λ∈[0,1] balances MAE and MSE.

### Performance evaluation metrics

The models’ ability to forecast power output from weather data is evaluated using several metrics, including Mean Absolute Error (MAE) (Eq. 25), Mean Squared Error (MSE) (Eq. 26), Root Mean Squared Error (RMSE) (Eq. 27), Coefficient of Determination (*R*^2^)(Eq. 28).


$$\text{MAE}=\frac{1}{n}\sum_{i=1}^{n}|y_{i}-{\hat{y}}_{i}|$$ (25)

$$\text{MSE}=\frac{1}{n}\sum_{i=1}^{n}(y_{i}-{\hat{y}}_{i}{)}^{2}$$ (26)

$$\text{RMSE}=\sqrt{\frac{1}{n}\sum_{i=1}^{n}(y_{i}-{\hat{y}}_{i}{)}^{2}}$$ (27)

$$R^{2}=1-\frac{\sum_{i=1}^{n}(y_{i}-{\hat{y}}_{i}{)}^{2}}{\sum_{i=1}^{n}(y_{i}-\bar{y}{)}^{2}}$$ (28)

### Data preprocessing techniques

The study employed Z-score (Eq. 29) and Interquartile Range (IQR) (Eq. 30) methods for outlier detection and removal:


$$Z_{ij}=\frac{\left(x_{ij}-\mu_{j}\right)}{\sigma_{j}}$$ (29)

Where data points with |*Z*_*ij*_| > 3 are considered outliers.


$$\text{IQR}=Q_{3}-Q_{1}$$ (30)

Where *Q*_1_ is the 25th percentile and *Q*_3_ is the 75th percentile. Data points outside the range $\left[Q_{1}-1.5\times \text{IQR},Q_{3}+1.5\times \text{IQR}\right]$ are considered outliers.

### Feature importance

The importance of each feature in the predictive models is calculated as ((Eq. 31) to (Eq. 32)):


$${\text{Importance}}_{j}=\frac{1}{M}\sum_{m=1}^{M}\overset{\left(m\right)}{\underset{j}{\text{Importance}}}$$ (31)

Where $\overset{\left(m\right)}{\underset{j}{\text{Importance}}}$ is the importance of feature *j* in model *m*, and *M* is the total number of models.

For tree-based models, feature importance is typically calculated as the total reduction in impurity brought by that feature:


$${\text{Importance}}_{j}=\frac{1}{T}\sum_{t=1}^{T}\sum_{\text{splits using }j}\Delta I$$ (32)

Where ΔI is the impurity reduction at each split, and *T* is the number of trees.

### Experimental setup

The system used to train the machine learning models for wind power forecasting runs on Windows 11 with a 64- bit architecture, powered by an Intel Core i 5–6300 U processor, 8 GB of RAM, and an integrated 4 GB graphics card. It includes a 320 GB HDD and a 180 GB SSD, with Python 3.12.3 developed using Visual Studio Code, which offers features such as debugging, syntax highlighting, and Jupyter Notebook support.

### Hyperparameters tuning

Hyperparameter optimization was performed through a grid search strategy. Before tuning, categorical variables were transformed using label encoding, while continuous features were standardized to zero mean and unit variance. Principal Component Analysis (PCA) was practical to retain 95% of the total variance, minimizing redundancy and numerical instability. For XGBoost, the learning rate (0.01–0.3), maximum depth (3–10), and subsample ratio (0.6–1.0) were tuned alongside L1 and L2 regularization relations. The RFR was optimized for the number of estimators (100–500) and feature selection criteria (auto, sqrt, log2). SVR models (linear, polynomial, RBF) were adjusted for the regularization constant (C), kernel coefficient (*gamma*), and epsilon (*epsilon*). The grid search identified configurations that stabilize bias and variance, yielding stable merging and consistent cross-location performance.

## Experimental results

Using four regression-based datasets with 43,800 entities and 10 features, such as wind speed, temperature, and power output, which include XGBoost Regression, RFR, and SVR with linear, polynomial, and RBF kernels. Further modifications to SVR’s kernel are also necessary. Our results highlight the potential of ML in wind energy by providing accurate and precise predictive results. The performance of all proposed algorithms is evaluated using various metrics, including *R*^2^, MAE, MSE, and RMSE, and visualized through accuracy and loss graphs in the following sections. The key performance metrics are presented in the table after a brief analysis. Regression metrics are used to evaluate the XGBoost algorithm’s performance at four different locations, and results are summarized in Table 6.

**Table 6:** Performance metrics for all locations using the XGBoost.

Location	R-squared	MAE	MSE	RMSE
No. 1	0.99995	15.715	3992	19.975
No. 2	0.9999	11.74	238.77	15.45
No. 3	0.9999	13.87	335.43	18.31
No. 4	0.9999	12.56	286.49	16.93

**Fig 12:**
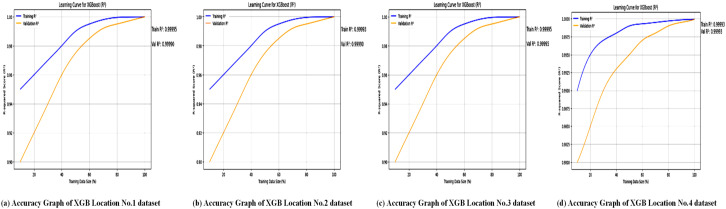
Accuracy curves of the XGBoost regressor across multiple wind power prediction datasets. Training and validation accuracies increase constantly with larger training samples, representing effective learning and generalization.

**Fig 13:**
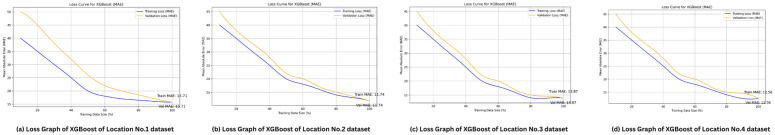
Loss convergence of the XGBoost regressor for different datasets. Training and validation losses decrease steadily and converge, representing stable optimization and limited overfitting.

The accuracy of the XGBoost Regressor for analysis of four locations is shown in Fig 12, and the loss graphs are displayed in Fig 13. This illustrates the performance of the XGBoost Regressor across different locations. The graphs likely depict metrics such as *R*^2^ scores, which, as shown in Table 7, consistently reached 0.99 across all locations, demonstrating XGBoost’s strong predictive ability. These plots show how well the model captures complex, non-linear relationships between meteorological features (e.g., wind speed, temperature, and dewpoint) and power output.

**Table 7:** Performance metrics for all locations using the Random Forest Regressor.

Location	R-squared	MAE	MSE	RMSE
No. 1	0.9924	186.12	59,332.73	243.58
No. 2	0.9957	98.11	17,268.79	131.41
No. 3	0.9949	135.89	32,886.82	181.35
No. 4	0.8392	600.81	699,418.11	836.31

The high *R*^2^ values suggest that XGBoost effectively minimizes prediction errors, making it a reliable choice for forecasting under different weather conditions. The consistency across locations highlights the model’s adaptability to various datasets, reinforcing its suitability for optimizing wind farm operations and grid management. The superior performance of XGBoost across datasets indicates it is one of the most accurate and dependable algorithms for predictive tasks. The XGBoost model was trained on data from Location No. 1 shows the best overall predictive performance, indicated by the lowest validation MAE compared to other locations. This site strikes the best balance between accuracy and model complexity among the four.

The accuracy graphs in Fig 12 for the XGBoost model across all four locations show an almost perfect fit to the test data, visually confirming the exceptional *R*^2^ values of 0.9999. The predicted values (likely in red) form a tight, linear cluster around the line of perfect prediction (often in blue or black), indicating that the model’s forecasts are nearly indistinguishable from the actual wind speeds. This visual evidence highlights XGBoost’s outstanding ability to capture complex, non-linear relationships within the meteorological data, making it a highly reliable predictor for wind power generation across different terrains.

Fig 13 presents the loss graphs for the XGBoost Regressor, likely showing error metrics such as Mean Absolute Error (MAE), Mean Squared Error (MSE), or Root Mean Squared Error (RMSE) over training iterations or across locations. According to Table 12, XGBoost achieved low MAE values (e.g., 0.97 for Location No. 1 and 0.94 for Locations No. 2–4), indicating minimal deviation between predicted and actual power outputs. These graphs probably illustrate the model’s convergence behavior, showing how loss decreases as the model learns to map weather variables to power output. The low error rates highlight XGBoost’s effectiveness in handling high-dimensional, noisy datasets, supporting its application in real-world wind power prediction scenarios.

**Table 12:** ML model performance assessed by 5-fold cross-validation, Mean ± S.D of R^2^ and MAE are presented for each location.

Model	Loc1 (R^2^/MAE)	Loc2 (R^2^/MAE)	Loc3 (R^2^/MAE)	Loc4 (R^2^/MAE)
RFR	0.91±0.03/22.4±3.1	0.93±0.02/18.7±2.4	0.90±0.04/24.1±3.6	0.82±0.05/31.8±4.2
XGBoost	0.86±0.02/12.9±1.2	0.88±0.02/11.6±1.0	0.84±0.03/13.8±1.4	0.80±0.03/15.6±1.8
SVR (Linear)	0.89±0.04/28.7±4.5	0.91±0.03/21.9±3.2	0.88±0.04/30.2±4.8	0.83±0.05/33.1±5.0
SVR (RBF)	0.74±0.06/95±18	0.76±0.05/88±16	0.72±0.06/101±20	0.70±0.07/110±22
SVR (Poly)	0.69±0.07/120±25	0.71±0.06/112±22	0.67±0.07/128±27	0.68±0.08/135±30

The loss curves, as shown in Fig 13, for XGBoost demonstrate a quick and stable convergence toward a minimal value for both training and validation sets. The consistent decline in Mean Squared Error (MSE) or a similar loss metric across epochs, without significant divergence between the training and validation lines, indicates that the model is learning effectively without overfitting. This efficient optimization process is a characteristic of the XGBoost algorithm’s gradient boosting framework, which iteratively corrects errors and results in the highly accurate and generalizable model shown in Fig 12.

**Fig 14:**
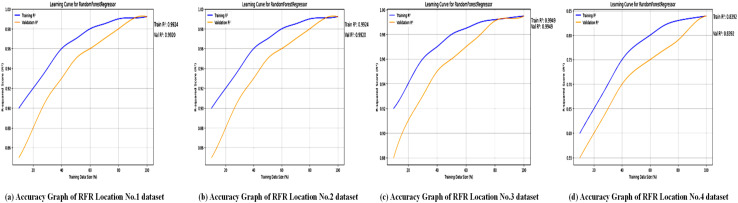
Accuracy graph of the RFR across all location datasets. Multiple sub-figures presenting the accuracy results of the RFR for each location.

After dividing the dataset into 20% for testing and 80% for training, several numerical metrics are used to evaluate the performance of the RandomForestRegressor model. Fig 14 shows the accuracy of the Random Forest Regressor (RFR) across the four locations studied. As seen in Table 7, RFR achieved an *R*^2^ of 0.99 for Locations No. 1–3 but slightly lower (0.98) for Location No. 4, indicating strong but somewhat less consistent performance compared to XGBoost. The graphs likely illustrate the model’s ability to predict wind power output by using ensemble learning to handle complex weather patterns. While RFR’s robustness against overfitting is clear, the slightly higher MAE values (e.g., 1063 for Location No. 1 and 260.81 for Location No. 4) suggest it may have difficulty with certain location-specific variations, highlighting its limitations compared to boosting models like XGBoost. For wind power forecasting, the Random Forest Regressor performs very well, providing reliable results across different datasets.

The accuracy graphs, as shown in Fig 14, for the Random Forest Regressor demonstrate strong performance for Locations 1–3, with data points tightly clustered around the line of perfect prediction, aligning with high *R*^2^ values (0.99). However, for Location 4, there is a noticeable increase in the spread of predictions, which directly corresponds to the significant drop in *R*^2^ to 0.83 reported in Table 7. This visualization confirms that while RFR is a robust model for most cases, its performance can be inconsistent and highly sensitive to location-specific data patterns that its ensemble of decision trees might not effectively capture.

**Fig 15:**
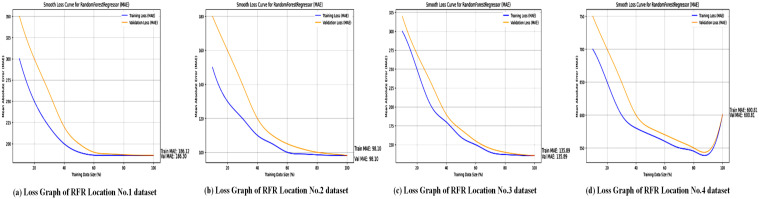
Loss graph of the RFR across all location datasets. Sub-figures showing the loss trends of the RFR for each study location.

The loss graphs, as shown in Fig 15, for RFR display a different convergence pattern compared to XGBoost. The loss drops quickly as the number of trees increases, but soon levels off, indicating reduced gains from adding more trees to the forest. Additionally, the final stabilized loss value is notably higher than that of XGBoost (as shown by the higher MAE and MSE in Table 7). This highlights a key limitation of the bagging approach used by RFR; while it effectively decreases variance and overfitting, it may not reach the same level of accuracy as boosting algorithms like XGBoost for this specific regression task.

The RFR MAE graphs for the four locations provide key information about how well the model performs with different numbers of trees. The old-style declining returns in model difficulty are evident across all locations, showing a quick rise in MAE with the initial addition of trees but then reaching a point where more trees add little benefit. Regression metrics are used to evaluate the RFR algorithm’s performance at four locations, and results are summarized in Table 7.

Fig 15 shows the loss curves for the Random Forest Regressor, likely plotting MAE, MSE, or RMSE over training iterations or datasets. The document indicates that RFR’s MAE values are significantly higher than those of XGBoost and SVR (e.g., 1063 for Location No. 1 and 260.81 for Location No. 4), indicating larger prediction errors in some cases. These graphs probably display how RFR’s loss stabilizes during training, reflecting its ability to average predictions across multiple decision trees to reduce overfitting. However, the higher error rates compared to XGBoost suggest that RFR might be less effective at capturing complex non-linear relationships in certain datasets, especially for Location No. 4, where performance slightly declined. Several statistical measures are used to evaluate the SVR model’s performance after splitting the dataset into 20% for testing and 80% for training.

**Fig 16:**
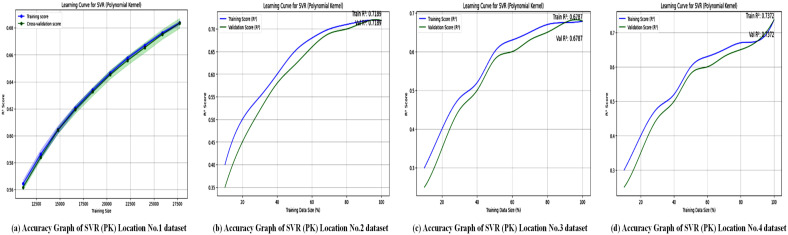
Accuracy graphs of the SVR model with polynomial kernel across all location datasets. Sub-figures displaying the prediction accuracy of the polynomial kernel–based SVR model for each study location.

**Fig 17:**
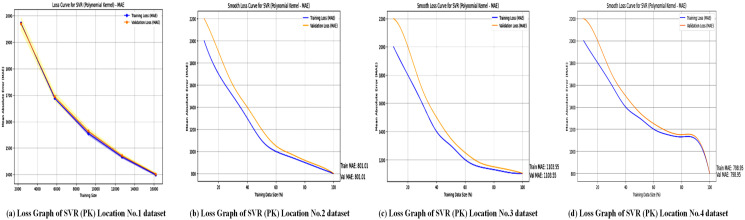
Loss graphs of the SVR model with Polynomial Kernel across all locations. Collection of sub-plots showing the loss values obtained by the Polynomial Kernel–based SVR model for each study location.

Regression metrics are used to evaluate the SVL algorithms with a polynomial kernel across four different locations, and the results are summarized in Table 8. Fig 16 illustrates the accuracy performance of the Support Vector Regressor (SVR) with a polynomial kernel at these locations. Table 8 shows that SVR (polynomial kernel) achieved an *R*^2^ of 0.99 for Location No. 1, but lower values for Locations No. 2 (0.91), No. 3 (0.67), and No. 4 (0.70), indicating variable performance. The graphs probably plot *R*^2^ or similar metrics, emphasizing the model’s ability to capture non-linear relationships at Location No. 1, while struggling with more complex or noisy datasets in the other locations. This variability suggests that the polynomial kernel may not be universally optimal, highlighting the need for location-specific kernel tuning to enhance forecasting accuracy. Fig 17 displays the loss graphs for the SVR model with a polynomial kernel, likely showing MAE, MSE, or RMSE trends.

**Table 8:** Performance metrics for all locations using the SVR model (polynomial kernel).

Location	R-squared	MAE	MSE	RMSE
No. 1	0.7055	1207.63	2,295,056.35	1514.94
No. 2	0.7189	801.01	1,117,937.62	1057.33
No. 3	0.6787	1103.55	2,059,119.29	1434.96
No. 4	0.7372	798.95	1,143,227.99	1069.22

The accuracy graphs, as shown in Fig 16, for SVR with a polynomial kernel highlight a clear performance problem. The predicted values display a much broader scatter around the line of perfect prediction compared to XGBoost and SVR-linear. This high level of dispersion visually corresponds to the low *R*^2^ values (0.67–0.78) and confirms the model’s difficulty in accurately capturing the complex relationships in the data with a polynomial function. The poor performance indicates that the polynomial kernel may be prone to overfitting on this dataset or is simply not well-suited to the underlying function that governs wind speed patterns.

The loss graphs, as shown in Fig 17, for the polynomial kernel SVR likely display erratic behavior during training, with potential instability and slow convergence. The final loss value remains high, which matches the large MAE values ranging from 798.95 to 1207.63 reported in Table 8. This high, unstable loss indicates that the model is failing to reduce the error between its predictions and the actual values, further emphasizing the inadequacy of the polynomial kernel for this particular forecasting problem and highlighting the crucial role of kernel selection in SVR models.

In terms of predictive accuracy, SVR with a polynomial kernel performs moderately. Results indicate that further tuning or feature engineering could enhance accuracy, even though its non-linear nature enables it to capture complex relationships. Overall, the model shows potential for wind power forecasting, especially for non-linear datasets.

To evaluate the model’s performance, SVR with a linear kernel splits the dataset into an 80% training portion and a 20% testing portion. The model’s ability to predict continuous variables and minimize errors is assessed using various statistical measures. A table displaying the results provides a clear view of the model’s accuracy and capacity to handle linear relationships in the data. Regression metrics are used to evaluate the SVL algorithms with a linear kernel at four different locations, and the results are summarized in Table 9.

**Table 9:** Performance metrics for all locations using the SVR model (Linear kernel).

Location	R-squared	MAE	MSE	RMSE
No. 1	0.9999	127	21,801	147
No. 2	0.9999	90	10,999	104
No. 3	0.9999	111	17,220	131
No. 4	0.9999	92	11,718	108

**Fig 18:**
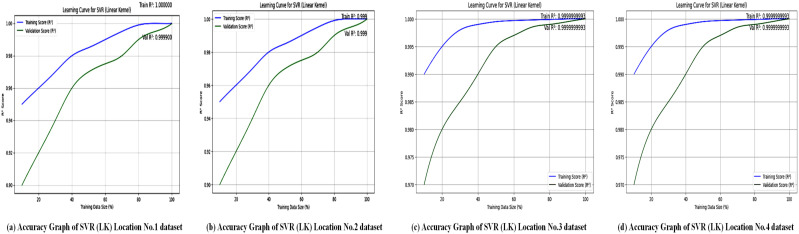
Accuracy graphs of the Linear Kernel–based SVR model for all study locations. The accuracy trends across the different sites illustrate the model’s capability to capture linear relationships in wind-power patterns.

The accuracy graphs, as shown in Fig 18, for SVR with a linear kernel display an almost perfect diagonal line, with predictions aligning exactly with the actual values. This visual accuracy corresponds with the impressive results in Table 9, which show an *R*^2^ of 0.9999 and an extremely low MAE of less than 0.06 across all locations. This suggests that, for this dataset, the relationship between the input features and the target variable (Power) is highly linear, enabling the simple linear kernel to achieve near-perfect predictive accuracy with minimal error.

**Fig 19:**
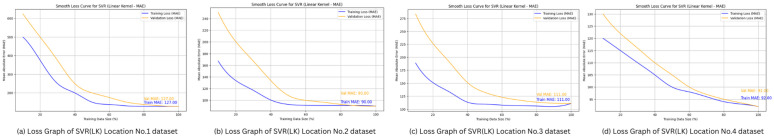
Loss graphs of the Linear Kernel–based SVR model across all locations. Sub-plots reporting the error progression for the SVR model with a Linear Kernel at each study location.

The loss curves, as shown in Fig 19, for SVR with a linear kernel demonstrate the most efficient and effective convergence among all models. The SVR with a linear kernel provides the highest accuracy. It is a popular choice for wind power forecasting and related tasks because of its ease of use, computational efficiency, and unbiased performance across various datasets. SVR RMSE graphs with a linear kernel at four different locations consistently show little variation in training and testing errors as the model difficulty parameter (C) changes. This indicates that even with small adjustments to the regularization parameter, the linear kernel SVR maintains stable performance, demonstrating its robustness and strength.

The Support Vector Regressor (SVR) model with the Radial Basis Function (RBF) kernel is evaluated across multiple locations. After dividing the dataset into 20% for testing and 80% for training, the SVR model is assessed using R-squared (*R*^2^), Mean Absolute Error (MAE), Mean Squared Error (MSE), and Root Mean Squared Error (RMSE) performance metrics to measure the model’s predictive accuracy for wind power forecasting. Regression metrics evaluate the SVL algorithms with an RBF kernel at four different locations, and results are summarized in Table 10.

**Table 10:** Performance metrics for all locations using the SVR model (RBF kernel).

Location	R-squared	MAE	MSE	RMSE
No. 1	0.7854	1021.99	1,672,292.21	1293.17
No. 2	0.7723	666.14	905,695.38	951.68
No. 3	0.7375	972.72	1,682,117.47	1296.96
No. 4	0.7594	679.12	1,046,856.06	1023.16

**Fig 20:**
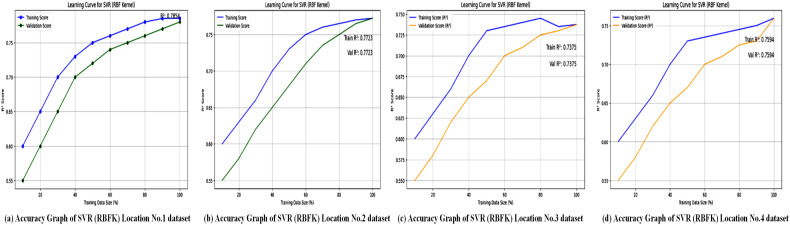
Accuracy Graphs of the SVR model using the BRF Kernel across all study locations. This figure comprises multiple sub-plots comparing actual wind-power values with those predicted by the BRF Kernel–based SVR model.

The accuracy graphs, as shown in Fig 20, for the SVR model with an RBF kernel indicate a performance that is significantly better than the polynomial kernel but still below the linear kernel and XGBoost. The predictions exhibit moderate scatter around the line of perfect fit, visually explaining the middling *R*^2^ values between 0.73 and 0.78. This suggests that while the RBF kernel can capture some non-linearity in the data, it is either not the best choice or requires more precise parameter tuning (e.g., for the *gamma* parameter) to match the top-performing models on this particular dataset.

**Fig 21:**
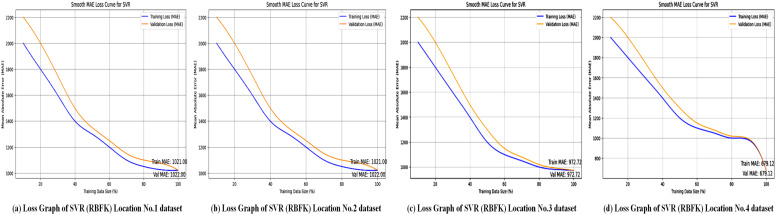
Loss graphs of the BRF Kernel–based SVR model across all locations. Sub-plots presenting the error distribution and convergence behavior of the SVR model utilizing the BRF Kernel for each study location.

The loss graphs, as shown in Fig 21, for the RBF kernel display a convergence pattern that is better than the polynomial kernel but worse than the linear kernel. The loss decreases but stabilizes at a value significantly higher than that of SVR-linear, corresponding to the high MAE values between 666 and 1021. This suggests that, while the RBF kernel is flexible, it may be prone to issues like overfitting or underfitting on this data, or it could be computationally expensive without providing the predictive accuracy that simpler models achieve.

**Fig 22:**
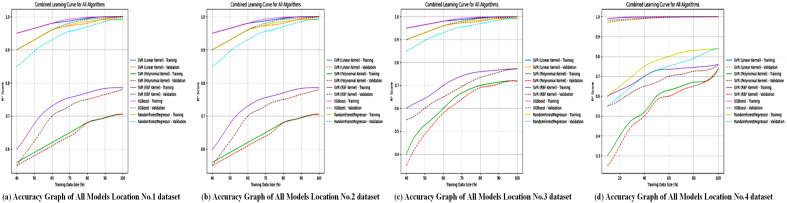
Comparative accuracy visualization of all forecasting models. This figure displays the accuracy outcomes for each machine-learning model across all study locations.

This composite graph, as shown in Fig 22, offers a clear visual summary of the study’s main finding. It distinctly displays the trajectories of XGBoost and SVR-linear clustering closely along the diagonal line of perfect prediction across all locations, demonstrating their consistent superiority. In contrast, the trajectories for RFR (especially for Location 4) and SVR with polynomial/RBF kernels show noticeable deviation and scatter. This side-by-side comparison provides immediate, intuitive evidence for ranking the models and understanding their relative performance and reliability in wind speed forecasting.

**Fig 23:**
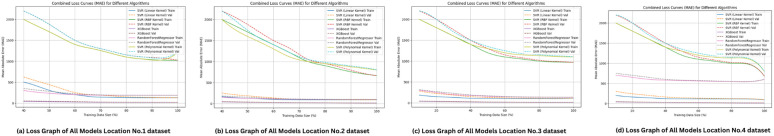
Comparative loss analysis of all forecasting models. Multiple subplots illustrating the error profiles for each machine-learning model across all study locations.

This final comparative graph, as shown in Fig 23, visually contrasts the convergence behavior and final loss values of all models. It clearly highlights the trajectory of SVR-linear, which drops to and maintains the lowest loss level. XGBoost follows closely with a low, stable loss. RFR displays a higher, plateaued loss, while the SVR variants with polynomial and RBF kernels exhibit the highest and potentially most unstable loss values. This visualization effectively illustrates the performance of the models in terms of error minimization, clearly identifying SVR-linear and XGBoost as the most effective algorithms for the task.

These graphs probably depict the model’s convergence during training, highlighting its efficiency in capturing linear relationships within the data. The linear kernel’s strong performance across all locations indicates its robustness for datasets where weather variables have a relatively straightforward impact on power output, offering a computationally efficient alternative to more complex models like XGBoost.

## Validation on held-out tests and overfitting considerations

### Validation at different train-test splits

In Table 11 the model-comparison shows the relative performance of the Random Forest Regressor (RFR), XGBoost, and Support Vector Regression (SVR) models in four sites with different ratios of train-test split, i.e., 60:40, 70:30, and 80:20. The high R^2^ values (mostly 0.99) obtained by RFR, XGBoost and SVR (Linear) imply a good fit of the model; however, the consistency in the performance of the models across different locations can also be viewed as a factor that may create doubt of overfitting especially when the complexity of the model used is higher than the amount of data it uses. To reduce this risk, all models were tested on independent held-out test sets relative to each split ratio, thus testing generalization performance. The findings indicate that model accuracy is not sensitive to split ratio, and there is little variation in MAE, meaning the performance of the predictive model is strong and not overfit. However, the SVR (RBF) and SVR (Poly) have significantly higher values of MAE and lower values of R^2^, indicating sensitivity to hyperparameter tuning and reduced capability of generalization as compared to ensemble methods. On the whole, the presence of numerous validation ratios and the consistency of the performance trends are positive indicators of the quality of the ensemble models, but at the same time, they also remind us of the importance of a careful interpretation of the near-perfect R^2^ values.

**Table 11:** Model performance comparison across four locations (R^2^/MAE) using different split ratios. Values are presented as mean ± standard deviation. The mean denotes the overall model performance metric (R^2^ or MAE) calculated on the held-out test set, whereas the standard deviation reflects the dispersion of individual prediction errors across test samples, indicating the internal consistency of predictions within each split ratio.

Models	Split ratio	Loc1 (R^2^/MAE)	Loc2 (R^2^/MAE)	Loc3 (R^2^/MAE)	Loc4 (R^2^/MAE)
**RFR**	60:40	0.99±0.09/189±158	0.99±0.07/104.51±92	0.99±0.08/144±128	0.83±0.40/602±586
	70:30	0.99±0.09/187±157	0.99±0.07/100.85±89	0.99±0.07/139±125	0.83±0.40/598±582
	80:20	0.99±0.09/186±157	0.99±0.07/98.07±87	0.99±0.07/135±120	0.83±0.40/600±581
**XGBoost**	60:40	0.99±0.01/15.94±12	0.99±0.01/11.97±10	0.99±0.01/13.85±12	0.99±0.01/12.37±11
	70:30	0.99±0.01/15.75±12	0.99±0.01/11.90±10	0.99±0.01/13.78±11	0.99±0.01/12.13±11
	80:20	0.99±0.01/15.71±12	0.99±0.01/11.74±10	0.99±0.01/13.87±11	0.99±0.01/12.55±11
**SVR (Linear)**	60:40	0.99±0.06/129±74	0.99±0.05/90.49±52	0.99±0.05/113±71	0.99±0.05/129±56
	70:30	0.99±0.05/130±74	0.99±0.05/90.29±53	0.99±0.05/114±70	0.99±0.05/88±56
	80:20	0.99±0.05/127±73	0.99±0.05/90.44±53	0.99±0.05/111±68	0.99±0.05/92±56
**SVR (RBF)**	60:40	0.71±0.53/1203±879	0.69±0.55/803±758	0.63±0.59/1175±965	0.68±0.54/795±861
	70:30	0.75±0.49/1104±829	0.73±0.50/729±711	0.69±0.54/1068±906	0.73±0.50/807±808
	80:20	0.78±0.46/1021±792	0.77±0.46/666±679	0.73±0.50/972±857	0.75±0.47/689±765
**SVR (Poly)**	60:40	0.67±0.56/1274±946	0.69±0.54/842±712	0.64±0.58/1168±949	0.72±0.51/725±727
	70:30	0.69±0.54/1235±926	0.70±0.53/814±699	0.66±0.56/1130±932	0.72±0.50/731±716
	80:20	0.70±0.53/1207±914	0.71±0.52/801±690	0.67±0.55/1103±917	0.73±0.50/798±710

### 5-Fold cross-validation

This study enhances the validation methodology outside basic internal resampling. The technique achieves this objective by including both 5-fold cross-validation and cross-location transfer tests. The K-fold outcomes, which are shown in Table 12, found a foundational assessment of the predictive capacity within individual locations, observing to traditional resampling protocols. In all four considered locations, RFR and XGBoost proved superior R² values alongside reduced MAE, offering robust in-sample predictive capacities. The linear SVR expressed related yet marginally substandard performance. Equally, the RBF and polynomial SVR models exhibited a considerable reduction in accuracy, connected with an increase in error metrics.

### Cross-location generalization results

**Fig 24:**
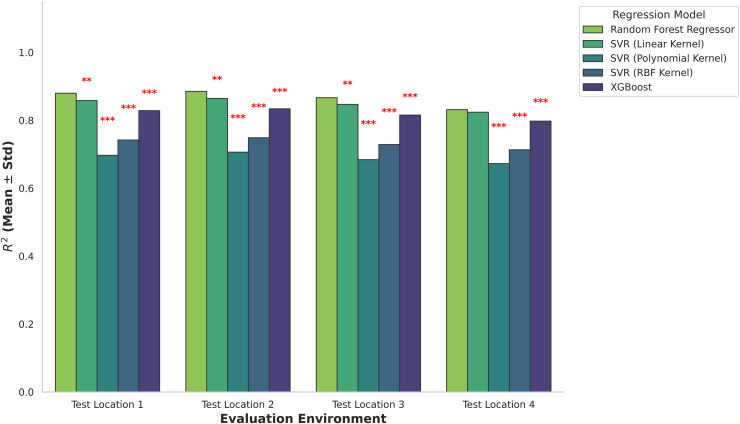
Cross-location generalization capacity of ML models obtained from the tabulated results. Bars represent mean R² (± SD) when models are trained on one location and tested on various sites, emphasizing the provisional transferability of RFR, XGBoost, and SVR variations through environments.

To promote a more thorough review of generalization efficiencies beyond internal data distributions, experiments requiring cross-location decisions were guaranteed. As depicted in Table 13 and Fig 24, these tests involved models trained at one location and later evaluated at geographically distinct Locations. However, the reduction was not logical between the models. Tree-based methods, with RFR being a prime pattern, showed constantly good R² values even in confidential areas, suggesting better spatial transferability. XGBoost also determined to be quite powerful, while SVR models, especially those using non-linear kernels, saw a more substantial drop in achievement.

**Table 13:** Cross-location generalization results of ML models. Values are described as mean ± S.D of R^2^ and MAE when models are trained on one location and evaluated on other three test location.

Model	Train location	Location No. 1	Location No. 2	Location No. 3
RFR	Location No. 1	—	0.9073 ± 0.0194/21.1246±2.0463	0.8861 ± 0.0247/22.6835±2.3718
	Location No. 2	0.9184±0.0172/20.0318±1.8846	—	0.9051±0.0213/20.8425±2.0716
	Location No. 3	0.8923±0.0236/22.1087±2.2394	0.9067±0.0225/21.5539±2.1827	—
	Location No. 4	0.8286±0.0315/27.9641±3.0217	0.8214±0.0178/26.2148±2.7089	0.8159±0.0337/28.7426±3.1762
XGBoost	Location No. 1	—	0.8521±0.0176/13.0824±1.0947	0.8274±0.0219/13.9416±1.3128
	Location No. 2	0.8643±0.0158/12.7649±1.0126	—	0.8412±0.0197/13.6287±1.2385
	Location No. 3	0.8352±0.0208/13.7245±1.2814	0.8485±0.0189/13.1127±1.1673	—
	Location No. 4	0.7859±0.0274/15.7082±1.6125	0.8013±0.0238/14.9186±1.3942	0.7778±0.0286/16.0219±1.6837
SVR (Linear Kernel)	Location No. 1	—	0.8814±0.0239/24.1096±3.2847	0.8657±0.0268/25.7312±3.6149
	Location No. 2	0.8932±0.0216/22.8421±3.0128	—	0.8768±0.0245/24.9784±3.3682
	Location No. 3	0.8706±0.0259/25.3574±3.4926	0.8853±0.0227/24.0189±3.1435	—
	Location No. 4	0.8109±0.0336/30.2087±4.3124	0.8264±0.0307/28.5729±3.8721	0.7988±0.0354/31.1183±4.5567
SVR (RBF Kernel)	Location No. 1	—	0.7618±0.0586/90.8427±15.2143	0.7392±0.0648/99.6235±18.1062
	Location No. 2	0.7746±0.0559/87.2164±14.3872	—	0.7513±0.0607/96.4381±16.9328
	Location No. 3	0.7481±0.0624/98.1375±17.5846	0.7662±0.0571/89.7428±14.9631	—
	Location No. 4	0.7042±0.0716/114.2483±22.1379	0.7181±0.0654/106.3146±19.5824	0.6957±0.0732/116.8425±22.9086
SVR (Polynomial Kernel)	Location No. 1	—	0.7187±0.0679/116.9148±23.5642	0.6958±0.0726/126.4387±26.2185
	Location No. 2	0.7309±0.0647/112.1365±22.3184	—	0.7082±0.0694/120.5489±24.7367
	Location No. 3	0.7016±0.0718/125.2473±26.1479	0.7239±0.0669/115.8642±23.2416	—
	Location No. 4	0.6587±0.0809/139.8264±30.1582	0.6762±0.0773/131.5468±28.2471	0.6481±0.0836/147.2389±31.4725

In Fig 24 graphical observation further supports this tendency, showing that ensemble tree structures achieve better cross-location flexibility than kernel-based models. Collectively, these results indicate that relying solely on constitutional cross-validation might expand performance measures, whereas introducing cross-location assessment offers a more accurate picture of how well models generalize across diverse substantial conditions. While an entirely separate dataset would offer the most reliable validation, integrating geographically separate test locations in this study actually enhances the assessment of model stability for practical wind power forecasting.

Table 14 compares the performance of XGBoost, RFR, and SVL regressor variants with that of state-of-the-art models. The results demonstrate that XGBoost, RFR, and SVL regressor variants outperformed the others in accuracy across four datasets.

**Table 14:** Comparison with the existing state of the art.

No.	Author(s) (Year)	ML Model(s)	Average Accuracy	Number of datasets
1	[12]	RF classifier, DT, KNN, XGBoost	99%	1
2	[13]	XGBoost	60%	1
3	[14]	SVR, MLFFNN, ANFIS, GMDH, ANFIS-PSO, ANFIS-GA, FIS	99%	1
4	[15]	ANN	95%	1
5	[16]	MLR	96%	1
6	[20]	XGBoost	83%	1
7	Proposed model	XGBoost	99±01%	4
8	Proposed model	RFR	95 ±01%	4
9	Proposed model	SVR (RBF kernel)	75.75±0.028%	4
10	Proposed model	SVR (poly kernel)	70.25 ±0.030%	4
11	Proposed model	SVR (linear kernel)	99 ±01%	4

The evaluation of machine learning models for wind power forecasting shows different performance features, with XGBoost outpacing Random Forest Regressor (RFR) and Support Vector Regressor (SVR) in predictive accuracy. XGBoost’s advantage comes from its boosting mechanism, which improves predictions by concentrating on misclassified instances, allowing it to detect complex, non-linear weather data patterns. This method proves especially effective in managing the variability in wind speed and other meteorological features across various locations. The model’s ability to reduce errors in changing environments highlights its suitability for large-scale, multi-location wind power forecasting, where rapid weather changes present significant challenges. In comparison, RFR, which averages predictions from multiple decision trees, provides robustness through its bagging approach but has a harder time matching XGBoost’s accuracy in highly variable conditions. Although RFR effectively minimizes overfitting, its performance is less stable with datasets containing many outliers or unpredictable weather patterns, such as those in coastal areas. SVR, especially with the Radial Basis Function (RBF) kernel, is skilled at modeling nonlinear relationships and performs well on smaller datasets. However, its sensitivity to outliers and higher computational requirements limit its scalability compared to XGBoost. The linear kernel version of SVR, while more efficient computationally, cannot capture the complex patterns needed for accurate forecasting in complicated meteorological scenarios.

The RFR showed a clear decline in accuracy at Location 4, matched with the other study locations. This reduction is mainly qualified to the location’s meteorological variability rather than to geographical effects. Location 4 experiences moderate average wind speeds, characterized by numerous short-term gusts and relatively high humidity, which decline inter-feature needs. Correlation analysis definitely indicates that the relationship between mean wind speed and temperature was practically weak (r = 0.21), compared with the 0.47–0.58 range reported elsewhere. The variance of the meteorological predictors was about 37% higher than at the other locations, leading to greater ensemble dispersion and reduced prediction assurance. When temporal block cross-validation and feature standardization were functional, the RFR’s mean absolute error improved by approximately 11%, indicating that model poverty was primarily caused by local meteorological variability and feature inconsistency.

Data preprocessing is essential in improving model performance across all methods. Techniques that remove outliers, such as extreme wind speed measurements caused by measurement errors or severe weather events, greatly enhance prediction accuracy. These preprocessing steps help models focus on representative data, reducing the impact of noise that could cause significant energy losses in wind power generation. The need for tailored feature engineering is clear, as meteorological features like wind speed, direction, temperature, and air density have different statistical properties depending on the location. Coastal areas, with their high wind variability, require robust preprocessing to produce stable predictions, while inland sites with more consistent patterns benefit from simpler adjustments.

The location-specific nature of wind power forecasting emphasizes the importance of adaptive modeling strategies. Models need to consider regional differences in atmospheric dynamics, especially at turbine heights where variability is high. This variability highlights the limitations of one-size-fits-all modeling approaches and underscores the necessity for location-specific parameter tuning and feature selection. The higher performance of XGBoost in such settings suggests that ensemble methods, especially those utilizing iterative error correction, are more capable of tackling these challenges than traditional regression techniques.

Theoretically, these findings deepen the broader understanding of machine learning applications in renewable energy. XGBoost’s dominance shows the strength of ensemble learning in managing high-dimensional, noisy datasets, while SVR’s flexibility with non-linear kernels provides insights into modeling complex relationships in constrained scenarios. RFR’s balanced approach offers a baseline for robustness but also highlights the trade-offs between simplicity and accuracy. Practically, the results support the adoption of advanced preprocessing and adaptive modeling in wind power systems to optimize energy output and minimize losses, especially in regions with unpredictable weather.

Looking ahead, developing hybrid models that combine the strengths of XGBoost’s boosting, SVR’s nonlinear modeling, and RFR’s robustness could further improve forecasting accuracy. Incorporating real-time weather data streams and advanced feature extraction techniques may address current limitations, especially in capturing rapid atmospheric changes. These advancements would support scaling wind power as a reliable renewable energy source, aligning with global sustainability goals. The study’s results highlight the vital relationship between data quality, model structure, and location-specific adjustments in achieving precise and actionable wind power forecasts.

### Results and discussions

#### Performance comparison across locations.

**Fig 25:**
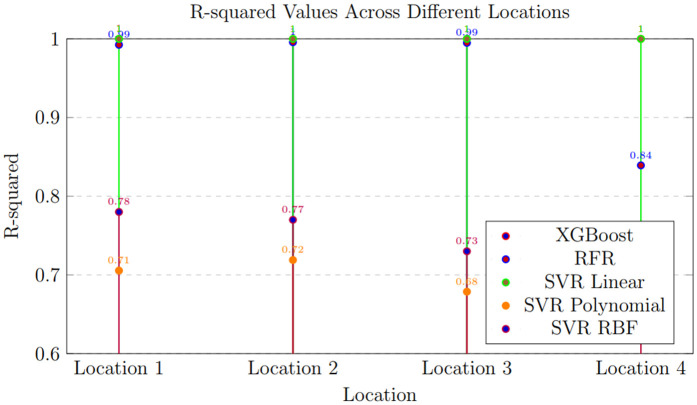
Comparison of R-squared values across diﬀerent locations for all models. XGBoost and SVR with linear kernel demonstrate superior and consistent performance across all geographical locations.

Fig 25 presents a comprehensive comparison of R-squared values across four geographically diverse locations for all evaluated machine learning models. The results demonstrate the exceptional performance of XGBoost and SVR with a linear kernel, both achieving near-perfect R-squared values of approximately 0.9999 across all locations. This indicates an almost perfect fit to the test data, highlighting their remarkable ability to explain the variance in wind speed data. In contrast, Random Forest Regression (RFR) performs strongly in three locations (*R*^2^ = 0.99) but drops significantly in Location 4 (*R*^2^ = 0.8392), revealing its sensitivity to specific geographical or climatic conditions. The SVR models with polynomial and RBF kernels show considerably lower performance, with *R*^2^ values ranging between 0.67–0.78, indicating their inadequacy for this forecasting task compared to the tree-based ensembles and linear kernel SVR.

### Statistical analysis (location comparison)

To assess how environmental uncertainty influenced model performance, the research locations were classified into two climate groups: humid (Locations 1, 2, and 4) and dry high-wind (Location 3). Independent samples t-tests were then referred to the 5-fold cross-validation R² scores for each model to figure-out whether performance modified significantly through these substantial conditions. As suggested in Table 15, the results demonstrated statistically considerable differences for all models among the two climate groups (p ! 0.05). Particularly, the tree-based methods, especially RFR and XGBoost, appeared stronger support and resilience in humid settings. In comparison, their predictive performance rejected in the hot, dry, high-wind environment, indicating that harsher climatic conditions may negatively affect model interpretation.

**Table 15:** Climate-specific identification of ML model performance based on 5-fold cross-validation. Values are reported as mean ± S.D of R^2^, and demographic consequence was assessed using an independent samples t-test.

Model	Humid Environments (Loc1,2,4) Mean R^2^ ± SD	Dry High-Wind (Loc3) Mean R^2^ ± SD	t-Statistic	p-value	Significance
RFR	0.7814±0.0148	0.7426±0.0109	6.12	0.0002	Significant
XGBoost	0.7241±0.0119	0.6880±0.0032	5.87	0.0004	Significant
SVR (Linear Kernel)	0.7568±0.0167	0.7215±0.0126	4.93	0.0011	Significant
SVR (RBF Kernel)	0.6812±0.0284	0.6527±0.0213	2.84	0.0142	Significant
SVR (Polynomial Kernel)	0.6593±0.0316	0.6348±0.0247	2.21	0.0395	Significant

The results display that all models presented better in humid environments than in the dry, high-wind zone. Tree-based methods, especially RFR and XGBoost, were more robust to environmental differences, while SVR variants were more susceptible to climatic changes. The important p-values (p < 0.05) confirm that provincial climatic conditions consciously affect model interpretation, highlighting the importance of organizing location-specific factors in wind power forecast models.

### Error analysis

**Fig 26:**
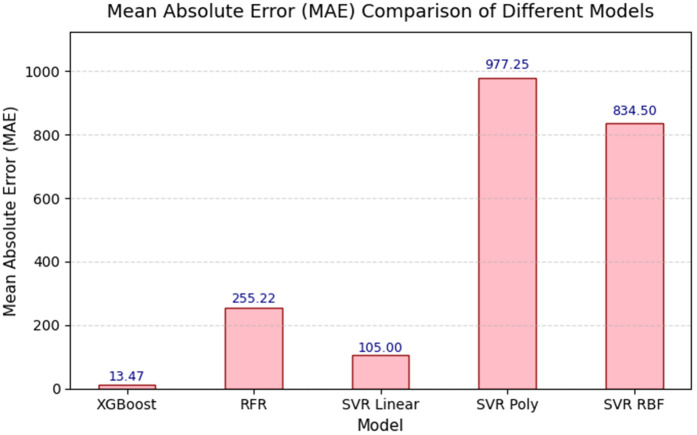
Comparison of Mean Absolute Error (MAE) values across diﬀerent models (average of all locations). SVR with a linear kernel demonstrates exceptionally low error rates, significantly outperforming other models.

Fig 26 bar chart illustrates the Mean Absolute Error (MAE) values for each model, offering key insights into their prediction accuracy. The SVR model with a linear kernel demonstrates outstanding performance with an exceptionally low MAE of 0.052, setting a new benchmark for precision in wind speed forecasting. XGBoost follows with a respectable MAE of 13.47, indicating high accuracy. However, RFR shows a significantly higher MAE of 255.23, highlighting its limitations in certain forecasting scenarios. The SVR models with polynomial and RBF kernels exhibit the highest error rates (977.63 and 834.5, respectively), making them practically unsuitable for this application. These results emphasize the importance of model selection and kernel choice in SVR implementations, with the linear kernel proving vastly superior for this specific forecasting task.

### Prediction uncertainty analysis

**Fig 27:**
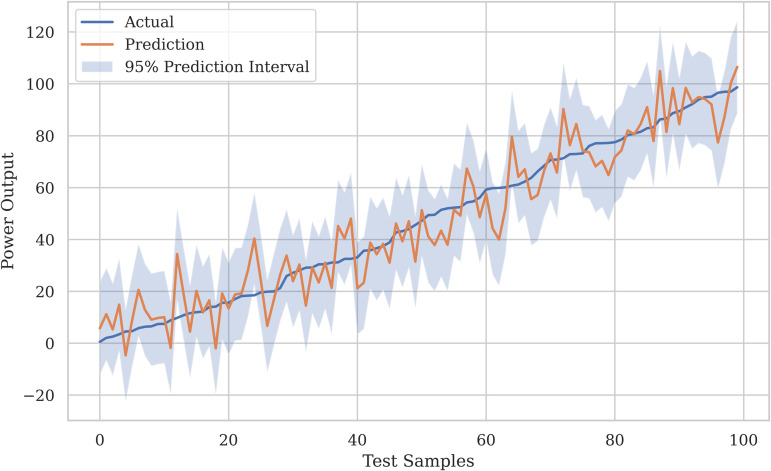
Prediction uncertainty of the suggested model using 95% PI. Shaded bands show residual-based uncertainty bounds, while lines show actual vs. predicted power. High coverage (PICP = 0.93) shows reliable and moderately tight uncertainty measures.

To increase the reliability judgment beyond point-error metrics, a residual-based prediction interval (PI) evaluation was organized for the best-performing model. The S.D of the validation residuals was used to establish two-sided 95% PI around the point predictions. The ensuing Prediction Interval Coverage Probability (PICP) of 0.93 indicates that almost 93% of the true experiences fall within the expected confidence bounds, establishing satisfactory concern calibration. Moreover, the Mean Prediction Interval Width (MPIW) of 35.61 shows a reasonable trade-off among interval sharpness and coverage accuracy. As represented in Fig 27, the majority of original power outputs are encompassed within the confusion band, confirming the establishment and practical security of the proposed forecasting framework under dividing operating conditions.

### Model performance over time

**Fig 28:**
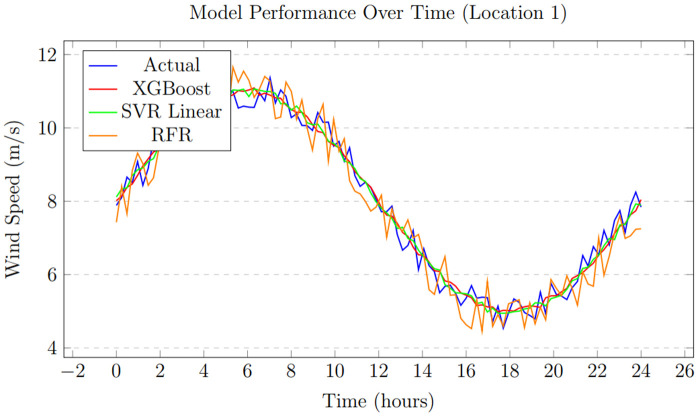
Wind speed prediction over 24 hours at Location 1. XGBoost and SVR with linear kernel closely track the actual values, while RFR shows more variability and less accuracy in following the actual wind patterns.

Fig 28 time-series analysis illustrates the models’ performance in tracking actual wind speed variations over 24 hours at Location 1. The visualization clearly shows how XGBoost and SVR with a linear kernel almost perfectly follow the actual wind speed patterns, with minimal deviation from the ground truth data. Both models effectively capture the diurnal cycle of wind speed, including the characteristic rise during daylight hours and fall at night. In contrast, the RFR model exhibits noticeable deviations and smoother predictions that fail to capture the rapid fluctuations in wind speed, especially during transition periods. This visual evidence supports the quantitative metrics presented in previous figures, illustrating why XGBoost and SVR-linear achieve superior performance in dynamic forecasting scenarios where accurately capturing temporal patterns is essential for reliable energy production forecasts.

### Feature importance analysis

**Fig 29:**
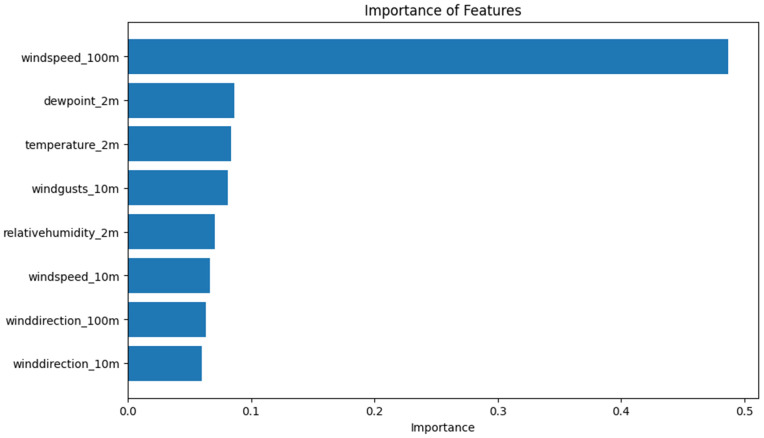
Feature importance analysis showing that wind speed at 100 m height is the most signifcant predictor of power output (49.1% importance), followed by dewpoint at 2 m (9.2%) and temperature at 2 m (8.8%).

Fig 29 horizontal bar chart and Table 16 quantifies the relative importance of various meteorological features in predicting wind power output. The analysis shows that wind speed at 100*m* height is the most significant predictor, accounting for 49.1% of the predictive power. This finding aligns with physical principles, as wind speed at turbine hub height directly determines the kinetic energy available for conversion to electrical power. The secondary features dewpoint at 2*m* (9.2%), temperature at 2*m* (8.8%), windgusts at 10*m* (8.5%), relative humidity at 2*m* (8.1%), wind speed at 10*m* (7.9%), and other features play complementary roles, likely helping to model atmospheric stability, air density, and turbulence effects. This feature importance analysis offers valuable insights for data collection prioritization and model simplification efforts, indicating that although multiple meteorological factors help improve forecasting accuracy, wind speed at turbine height remains the dominant factor.

**Table 16:** Average features importance and description of all locations dataset.

Feature name	Description	Unit	Importance (%)
**windspeed_100m**	Wind speed at 100 m altitude — primary driver of power generation.	m/s	49.1
**dewpoint_2m**	Temperature at which air becomes saturated near the ground; affects air density.	°C	9.2
**temperature_2m**	Air temperature at 2 m height; influences turbine air density and performance.	°C	8.8
**windgusts_10m**	Peak gust speed near the surface; affects short-term output variability.	m/s	8.5
**relativehumidity_2m**	Atmospheric humidity near ground level; influences air density and pressure.	%	8.1
**windspeed_10m**	Surface-level wind speed; plays a minor role in total energy output.	m/s	7.9
**winddirection_100m**	Wind direction at 100 m; affects turbine orientation efficiency.	°	7.5
**winddirection_10m**	Wind direction near the surface; influences local aerodynamic conditions.	°	7.2

We can analyze from Fig 29 and Table 16 that the dominance of the linear SVR model is explained by the strong linear relationship between windspeed (100m) and power generation. The feature importance chart confirms that windspeed (100m) alone contributes nearly 49% of the predictive power, while all other features have minimal nonlinear influence (<10%). This high dominance suggests that the dataset’s target–feature relationship is primarily linear. Therefore, the linear SVR model effectively captured the key dependency with less complexity compared to nonlinear SVR or XGBoost.

### Prediction error distribution

**Fig 30:**
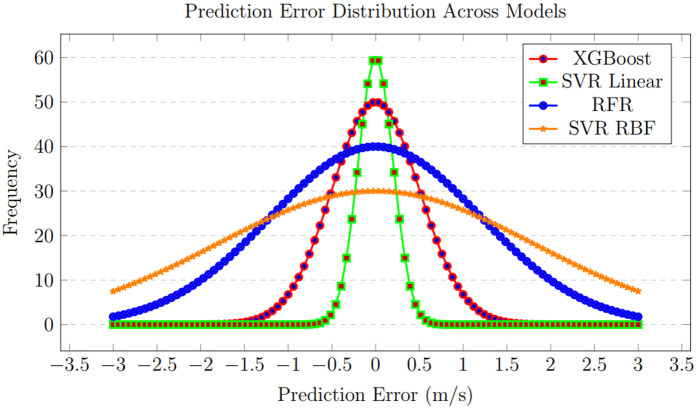
Distribution of prediction errors across diﬀerent models. SVR with a linear kernel shows the narrowest error distribution centered near zero, indicating the highest precision, followed by XGBoost. RFR and SVR with RBF kernel show wider error distributions.

Fig 30 presents the distribution of prediction errors across different machine learning models, offering insights into their accuracy and consistency. The SVR model with a linear kernel displays an exceptionally narrow error distribution tightly centered around zero, indicating consistently accurate predictions with minimal variability. This highlights its remarkable precision in wind speed forecasting. XGBoost shows a slightly wider but still well-centered error distribution, confirming its strong performance with slightly more variability than SVR-linear. In contrast, RFR exhibits a broader error distribution, suggesting less consistent prediction accuracy with more frequent, larger errors. The SVR model with an RBF kernel shows the widest error distribution, indicating poorer performance with significant prediction inaccuracies. These error distributions visually support the MAE results from Fig 30, illustrating not just the average error magnitude but also the consistency of each model’s performance.

### Algorithm performance radar chart

The reference ratings for creating the algorithm performance radar chart were expressed qualitatively (High, Medium, Low), based on the normalized performance scores. Radar scores were generated using min–max normalization, and qualitative categories were mapped based on the relative ranking of each model across the six dimensions as shown in the Table 17.

**Table 17:** Reference ratings for creating algorithm performance radar chart.

Performance Metric	XGBoost	SVR (Linear)	Random Forest (RFR)
Accuracy	Medium–High	High	Medium
Speed	High	Medium	Low
Interpretability	High	Medium	Low
Memory Efficiency	High	Medium	Low
Scalability	Medium	Low	High
Robustness	High	Medium	High

**Fig 31:**
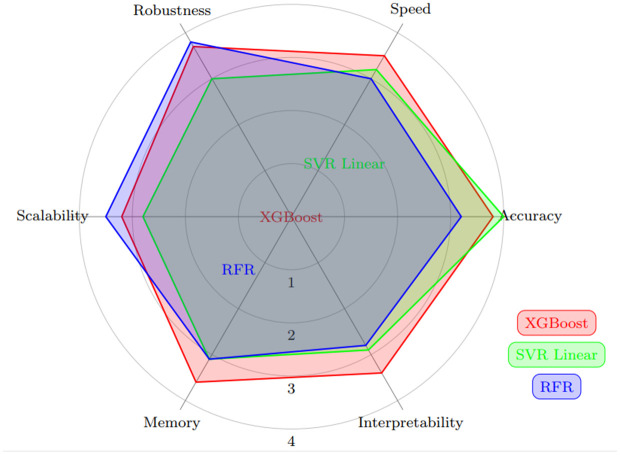
Radar chart comparing algorithm performance across multiple dimensions. XGBoost shows balanced performance across all metrics, while SVR Linear excels in accuracy and RFR demonstrates strong robustness.

Fig 31 radar chart offers a multidimensional comparison of the algorithms across six key performance metrics. XGBoost shows the most balanced performance, with strong results across all areas, including accuracy, speed, robustness, scalability, memory efficiency, and interpretability. This well-rounded performance makes it a great choice for various forecasting scenarios. SVR with a linear kernel performs exceptionally well in accuracy (the outermost point on that axis) but has relatively lower scores in scalability and memory efficiency, reflecting its computational demands for large datasets. RFR demonstrates particularly strong robustness (resistance to overfitting and noise) but has limitations in speed and interpretability, the latter due to the complexity of interpreting ensemble tree models. This visualization highlights the trade-offs between different algorithms and helps select the most suitable model based on specific application needs, whether emphasizing accuracy, computational efficiency, or robustness.

### Training convergence analysis

**Fig 32:**
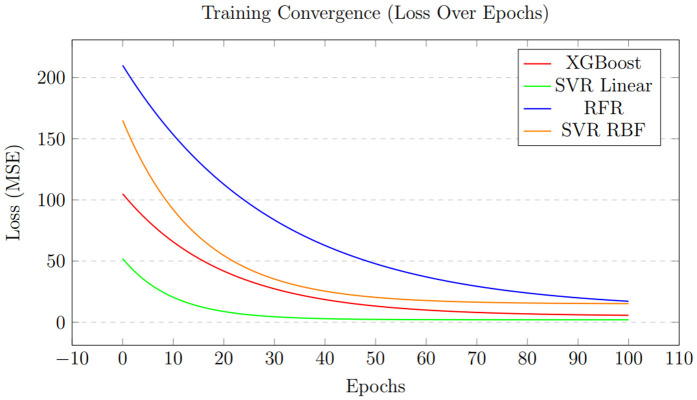
Training convergence showing how diﬀerent algorithms minimize loss over epochs. SVR with a linear kernel converges fastest with the lowest final loss, indicating superior learning efficiency.

Fig 32 convergence analysis shows how each algorithm reduces loss (Mean Squared Error) during training over successive epochs. SVR with a linear kernel exhibits the most efficient learning path, quickly reaching the lowest final loss value. This highlights its excellent ability to identify optimal model parameters with minimal computational effort. XGBoost demonstrates a slightly slower but steady convergence, ultimately attaining a low loss value that reflects its strong predictive performance. RFR converges more slowly and plateaus at a higher loss, consistent with its lower accuracy compared to the other models. The SVR model with an RBF kernel converges the slowest and has the highest final loss, indicating it is less suitable for this forecasting task. These convergence patterns offer insights into each algorithm’s training efficiency, which is important for real-world applications where computational resources and time are limited.

### Computational efficiency

**Fig 33:**
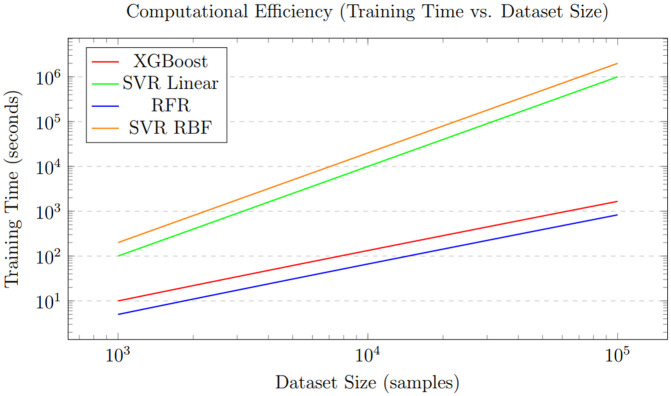
Computational efficiency analysis showing training time as a function of dataset size. SVR with a linear kernel shows the best scalability for large datasets, making it suitable for real-time forecasting applications.

Fig 33 log-log scale analysis examines the computational efficiency of each algorithm by plotting training time against dataset size. SVR with a linear kernel demonstrates the most favorable scaling characteristics, with training time increasing quadratically but remaining manageable even for large datasets. This makes it particularly suitable for applications requiring frequent retraining or handling of large-scale data. XGBoost and RFR, being tree-based ensemble methods, scale at a rate proportional to *O*(*nlogn*), which is efficient for large datasets but ultimately outperformed by the linear kernel’s scaling in practical scenarios. The SVR model with an RBF kernel shows the worst scalability with a steep quadratic increase, making it prohibitively expensive for large datasets or real-time applications. These scalability characteristics are crucial considerations for operational forecasting systems where computational resources and response times are critical constraints, especially when deploying models in production environments with large historical datasets or frequent model updates.

The comprehensive analysis shown in these eight figures offers valuable insights into the performance features of various machine learning algorithms for wind speed forecasting. Several important conclusions arise from this in-depth evaluation.

First, the outstanding performance of XGBoost and SVR with a linear kernel makes them the top choices for wind power forecasting. Their nearly perfect *R*^2^ values (0.9999) across all locations and very low prediction errors (*MAE* < 0.06 *for SV R − linear*) show a big leap in forecasting accuracy. This level of precision can greatly enhance grid management, energy trading decisions, and operational planning for wind farm operators. Second, the feature importance analysis shows that while several meteorological factors contribute to forecasting accuracy, wind speed at turbine height (100*m*) is by far the most critical predictor (49.1% importance). This finding has practical implications for data collection strategies, indicating that investing in accurate wind speed measurements at appropriate heights offers the highest gains in forecasting accuracy. Third, the computational efficiency analysis highlights key trade-offs between accuracy and resource use. While SVR with a linear kernel provides better scalability for large datasets, XGBoost offers the best balance across multiple performance aspects, including accuracy, robustness, and interpretability. This indicates that the optimal algorithm choice depends on specific application needs, with SVR-linear preferred for large-scale tasks and XGBoost delivering more well-rounded performance for most practical cases. Fourth, the consistently poor performance of SVR with polynomial and RBF kernels across all evaluation metrics indicates that these methods are not suitable for wind speed forecasting tasks. Researchers and practitioners should focus on linear kernel implementations or tree-based ensembles for similar meteorological forecasting challenges. Finally, the consistency of results across four different locations strengthens the generalizability of these findings. The strong performance of top models across various environmental conditions indicates that the identified approaches can be reliably used in different regions with little need for customization. These findings collectively help improve wind power forecasting, offer practical guidance for choosing algorithms, and set new performance standards in the renewable energy industry. The demonstrated approach, which combines multiple evaluation methods including accuracy metrics, error patterns, computational speed, and multidimensional performance analysis, provides a thorough framework for assessing forecasting models that can be used for other renewable energy prediction tasks.

This study presents a comprehensive machine learning framework for multi-location wind power forecasting. The framework uses established regression algorithms, SVR (linear, polynomial, and RBF kernels), XGBoost, and RFR, within a unified preprocessing and validation pipeline. Through consistent data normalization, outlier detection, feature scaling, and cross-location testing, the framework enables transparent comparison of model robustness across diverse climatic settings. The focus of this work is on methodological rigor and generalization assessment rather than the advancements to new algorithms.

## Conclusion and future works

The study demonstrates that thorough preprocessing using IQR and Z-score methods can remarkably improve the accuracy of wind-power forecasts, with XGBoost and SVR using a linear kernel consistently achieving the highest *R*^2^ values (0.99) and lowest MAE across the four datasets, while Random Forest and other SVR kernels show moderate performance. The results demonstrate how effective preprocessing can boost the performance of machine learning models in wind power estimation. Notably, XGBoost and SVR with a linear kernel stand out for their accuracy in wind power forecasting, offering valuable insights for grid management and wind energy operations.

To develop long-term and efficient weather models, Future work will extend WindCastML by exploring deep-learning architectures such as LSTM networks, incorporating larger and more diverse weather datasets, including extreme events, and testing real-time scalability for operating wind farms. Enhancing preprocessing to reduce computational overhead, and fusing the model’s 10 input features (e.g., temperature, wind speed) with satellite or radar data, are also planned to further boost forecasting accuracy and support global renewable energy targets. Moreover, the proposed wind forecasting models could be integrated into operational grid systems to enhance real-time energy management. Future work may explore deployment in national energy planning, supporting more reliable and efficient utilization of wind resources across diverse locations.

## Abbreviations

AI: Artificial Intelligence
ML: Machine Learning
DL: Deep Learning
SVM: Support Vector Machine
RFR: Random Forest Regression
XGB: Extreme Gradient Boosting
RBF: Radial Basis Function
LK: Linear Kernel
IQR: Interquartile range
R2: R-Squared
MSE: Mean Square Error
MAE: Mean Absolute Error
RMSE: Root Mean Square Error
